# Mobile Apps to Prevent Violence Against Women and Girls (VAWG): Systematic App Research and Content Analysis

**DOI:** 10.2196/66247

**Published:** 2025-06-23

**Authors:** Md Hafizur Rahman, Nasimul Ghani Usmani, Pradip Chandra, Ridwana Maher Manna, Anisuddin Ahmed, Mohammad Sohel Shomik, Shams El Arifeen, Aniqa Tasnim Hossain, Ahmed Ehsanur Rahman

**Affiliations:** 1 Maternal and Child Health Division, International Centre for Diarrhoeal Disease Research, Bangladesh, 68, Shaheed Tajuddin Ahmed Sarani, Dhaka, 1212, Bangladesh, 880 1878072838; 2 Nutrition Research Division, International Centre for Diarrhoeal Disease Research, Bangladesh, Dhaka, Bangladesh

**Keywords:** violence against women and girls, mHealth, mobile app, smartphone app, prevention, mobile health

## Abstract

**Background:**

Numerous reviews have explored specific aspects of violence prevention apps, but given the rapid development of new apps, increased violence during COVID-19, and gaps in understanding functionalities and geographical distribution, an updated review is needed.

**Objective:**

Therefore, we aimed to systematically evaluate the trends, geographical distribution, functional categories, available features, and feature evolution of mobile apps designed to prevent violence against women and girls (VAWG).

**Methods:**

We conducted a systematic search on app reselling platforms and search engines from April 24, 2024 to May 28, 2024, using terms related to VAWG in multiple languages. We included apps meeting our criteria for addressing VAWG, without restrictions on date or language. We conducted content analysis of app and apps were categorized by functionality and feature type. We performed descriptive analyses, trend analysis, co-occurrence network analysis, and geographical mapping.

**Results:**

Out of 432 apps initially identified, 178 were included in the final analysis. Of these, 99 apps were available on both Google Play and the App Store, and 64 were exclusive to Google Play. Most apps were implemented in North America (48/178, 27%), followed by South Asia (31/178, 17%) and Europe and Central Asia (31/178, 17%). Emergency and support apps were most prevalent across regions. Most apps (132/178, 74%) originated from the private sector and were designed for survivor (121/178, 68%), were free without in-app purchases (100/178, 56%), had a website (148/178, 83%), and offered GPS features (142/178, 80%), but only 15% (27/178) provided offline functionality. App releases peaked in 2020 (33/178, 19%), followed by a decline. Regression analysis indicated a significant trend (*P*=.01) increase in app release, with a 2.40 unit increase per year before 2020 and a 7.01 unit decrease after, showing a post-2020 decline of 4.61 units per year. Apps were primarily categorized as emergency (n=110) or support (n=81), with most emergency apps in the 10,000 to ≥100,000 downloads range. Network analysis showed that emergency services (degree=10, clustering coefficient=0.911), location sharing (degree=10, clustering coefficient=0.911), SOS (Save Our Souls) alerts (degree=10, clustering coefficient=0.911), and educational resources (degree=10, clustering coefficient=0.911) features highly co-occurred in the same app. We found a gradual shift towards more sophisticated and comprehensive safety tools, evolving from basic GPS tracking and SOS alerts to advanced features such as real-time communication, panic buttons, peer support, and group communication, culminating in multifunctional platforms offering personalized safety, community engagement, and proactive risk identification.

**Conclusions:**

Most apps to prevent VAWG emphasize emergency and support functions, and although initial releases increased, there has been a recent decline, with a shift towards integrating more comprehensive safety solutions such as communication, reporting, and community engagement. Future app development should prioritize cross-platform availability, offline functionality, public sector collaboration, and the integration of advanced technologies like artificial intelligence.

## Introduction

Violence against women and girls (VAWG) remains a pervasive global health issue, with profound physical, psychological, and social impacts. The statistics are harrowing: VAWG affects 1 in 3 women in their lifetime, 200 million women experienced genital manipulation, 7% of women experienced sexual assault by a nonpartner, 35% of women experienced physical or sexual violence, and 38% of murders of women are committed by intimate partners [1]. Every 11 minutes, a woman or girl is murdered by a family member or intimate partner, resulting in nearly 50,000 such deaths annually [2]. VAWG results in significant physical and mental health impacts, including injuries, psychological trauma, and an increase in chronic health issues. In addition, the economic costs are substantial, affecting health care systems, productivity, and families, with survivors bearing considerable medical and mental health care expenses [3].

Interventions to prevent VAWG have traditionally included a range of legal, social, community, and technology-based approaches. With the advent of the digital revolution, technology has significantly enhanced and expanded these interventions [4]. Digital technologies have shown immense potential to prevent VAWG [5]. The integration of digital technologies, particularly mobile apps, into VAWG prevention and response strategies represents a relatively nascent and rapidly evolving frontier. Mobile apps offer potential interventions ranging from emergency assistance to educational resources. Besides, mobile apps provide information, support, and intervention services to survivors of violence. These tools range from safety planning and assessment applications to platforms promoting behavior change among perpetrators [6-10]. As a result, mobile apps were found to be useful for women’s personal safety, preventing violence, and accessing support [10]. One study reported that mobile apps were perceived to be more effective than pepper spray in ensuring personal safety [11].

Between 2019 and 2022, numerous review studies analyzed mobile apps and focused on specific facets such as personal safety [6,10], antirape [7], domestic violence [12], and intimate partner violence [9]. Most previous reviews on mobile apps often focused on specific forms of violence and overlooked others. The first comprehensive review was conducted by Eisenhut et al [8] in August 2018, provided an initial analysis and functional categorization of mobile apps addressing VAWG through a systematic review . However, in 2024, approximately 1000 new apps are released daily on the Google Play Store alone [13]. The rapid development of new apps in recent years necessitates an updated review to capture recent innovations and emerging trends. Besides, during the COVID-19 pandemic in 2020, there was a significant increase in VAWG [14], underscoring the need to examine the trends in app usage and development during this period.

In addition, previous studies did not include detailed analyses of web-based or offline capabilities and the country or region-wide distribution of mobile app functionalities designed to prevent VAWG. Such knowledge is crucial for understanding key components of app functionalities and their distribution across countries, guiding the development of more targeted and effective interventions to prevent VAWG. Furthermore, previous studies also revealed a significant lack of extensive research on mobile apps intended to prevent VAWG [15,16]. Therefore, we aimed to systematically evaluate the trends, geographical distribution, functional categories, available features, and feature evolution of mobile apps designed to prevent VAWG.

## Methods

### Study Design and Search

To identify the apps, we conducted a systematic search on app reselling platforms and search engines between April 24, 2024, and May 28, 2024. The reselling platforms included Google Play Store, Apple App Store, and Microsoft Store. The search engines included Google, Bing, Baidu, Yandex, DuckDuckGo, Yahoo, Ecosia, and Ask.com. Key search terms included terms related to VAWG and its different forms. A detailed list of the search terms is provided in .

The search was conducted in multiple languages, including English, Spanish, French, Portuguese, Russian, Chinese, Bangla, German, Swedish, Japanese, Korean, Urdu, Hindi, Vietnamese, and Malay. Google Translator was used to translate English search terms into other languages to ensure coverage across different regions and languages. In addition, apps identified in previous systematic reviews up to May 2024 and still available on reselling platforms were also included. The app search was conducted jointly by 2 authors (NGU and PC) to ensure accuracy. We adhered to the PRISMA (Preferred Reporting Items for Systematic Reviews and Meta-Analyses) guidelines in constructing this review, as outlined in Checklist 1 [17]. A protocol was developed before conducting the review, and the protocol was registered in the International Prospective Register of Systematic Reviews (PROSPERO; CRD42024500431) and there were no deviations from the registered protocol.

### Eligibility Criteria

We included all mobile apps found and available on the specified web-based or platform-specific app stores, with no date and language restrictions. The inclusion criteria focused on apps specifically designed to address VAWG, available on Google Play Store, Apple App Store, Microsoft Store, or as Websites. Apps were required to have at least 1 functional feature directly related to the safety, support, or prevention of VAWG. We did not have any date, number of downloads, and language restrictions.

### Data Extraction

We conducted a content analysis of the app’s features. App content was extracted by installing the app on a mobile device. If an app was not freely available or purchase was required for installation or use, we purchased the app. Besides, contents were also extracted from the “about this app” section on the Google Play Store, the “app support” section on the Apple App Store and from the app’s corresponding website. Data extraction was conducted jointly by 2 authors (NGU and PC) to ensure accuracy and completeness. Microsoft Excel was used for the systematic management of app identification, screening, and data extraction.

A structured data extraction form in Excel was used to capture the following variables: app name, app details (description, functionality, and target audience), functional categories, country of initiation, region of initiation, country of implementation, region of implementation, owner institution name, developer institution name, year of first release, year of last update, number of downloads, number of ratings, overall ratings, sector (public, private, or both), target groups (survivors only, nonsurvivors only, or both), primary source of the app (reselling platform or search engine), availability on reselling platforms (web only, App Store, Google Play, Windows Store, or multiple platforms), cost (free without in-app purchases, free with in-app purchases, not free, and unclear), type of VAWG addressed, overall objective of the app, all features, GPS or navigation feature (yes, no), offline mode (yes, no), content language, website availability (yes, no), website link, and whether an external device is required (yes, no, and the name of the external device if applicable). Discrepancies in the app selection process, data extraction, and analysis were resolved through a secondary review by the authors and discussion among authors to ensure accuracy and consistency.

### Categorization

Apps were categorized into functional categories based on the functionality of features. The functional categories were adopted from a previous systematic review [8]. A detailed definition of functional categories is given in Multimedia Appendix 1an app fell into multiple functional categories, it was assigned to all relevant categories. The functional categories included:

1. Emergency response: Apps providing quick access to emergency contacts and services.
2. Educational: Apps offering information and resources on violence prevention and legal rights.
3. Support services: Apps connecting users to support groups, counseling, and helplines.
4. Reporting tools: Apps enabling users to report incidents of violence anonymously or directly to authorities.
5. Safety planning: Apps assisting in the creation of personalized safety plans.

In addition, features were classified according to their type as follows: emergency and safety alerts, location tracking and sharing, safety tips and guidance, communication and support, education and awareness, reporting and documentation, privacy and security measures, and community engagement and collaboration. If an app’s feature belongs to multiple feature types, the app was classified under all applicable types. Details of feature categories and corresponding features are given in Multimedia Appendix 1.

Furthermore, countries of initiation and implementation were categorized into regions according to World Bank regions [18]. Offline mode was defined as the app’s ability to operate without an internet connection. Cost was defined as follows:

1. “Free without in-app purchases” indicates the app is free with no additional costs.
2. “Free with in-app purchases” indicates the app is free to download but includes optional purchases.
3. “Not free” indicates the app requires payment for download.
4. “Unclear” indicates insufficient information to determine the cost structure.

### Analysis

The analysis of mobile app release trends was conducted using the R (R Core Team) programming language [19], using a structured statistical approach to evaluate temporal patterns from 2010 to May 2024. We began by compiling annual frequency data, which was organized into a data frame for systematic analysis. To visualize these trends, a line plot was created using the *“ggplot2”* package [20], with data points and labels included to enhance interpretability. Cumulative frequency analysis was performed using *“ggplot2”* to illustrate the overall growth trajectory of app releases over time. We used linear regression modeling using *“ggplot2”* to quantify the relationship between the year and the number of app releases, assessing statistical significance through the *P* value of the regression slope. The regression line and CIs were added to the plot to depict model fit and variability. In addition, segmented regression analysis and plotting were conducted using the “*segmented*” package [21], with a predefined breakpoint in 2020, to identify potential changes in the rate of app releases before and after the onset of the COVID-19 pandemic.

In addition, using the *“igraph”* [22] and *“tidyverse”* [23] packages in R, we constructed and visualized a co-occurrence network to analyze features that are mostly present together in an app. A co-occurrence matrix was developed to represent the frequency of feature pairings, which was subsequently transformed into an *“igraph”* object for network analysis. In the visualization, nodes denoted features, and edges signified the weighted frequency of co-occurrences, with node sizes scaled by the degree to indicate prominence. The network was plotted using the Fruchterman-Reingold layout for enhanced visual separation [24]. Besides, we computed key network statistics, including node degree, betweenness centrality, closeness centrality, eigenvector centrality, clustering coefficient, average path length, and network density, to assess the structural properties and significance of features within the network Multimedia Appendix 1.

Furthermore, we conducted descriptive analyses using Microsoft Excel for app availability on reselling platforms, feature types, and the most prevalent (features that were available more than once) features by year and percent distribution of sectors, target groups, cost, website availability, GPS functionality, and offline mode. Besides, we analyzed the overall frequency distribution of functional categories, as well as the distributions by sector, number of downloads, and GPS functionality. In functional category plotting, responses were categorized as multicategory classification to account for instances where apps could be classified under more than one functional category. Geographical maps were developed by Adobe Illustrator to present the regional distribution of maps and their functional categories.

## Results

### App and Reselling Platform

**Figure 1.**
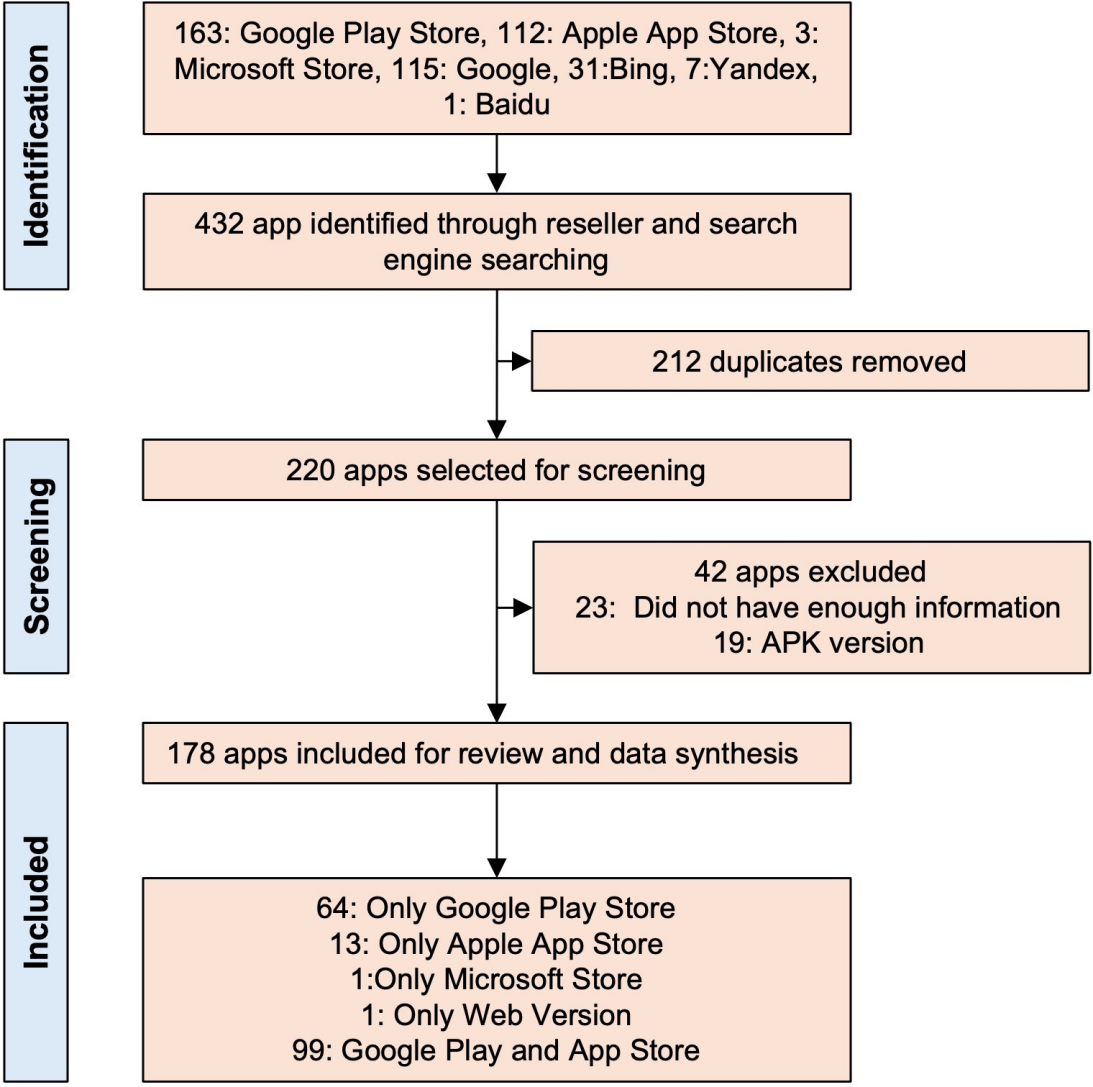
ofPreferred Reporting Items for Systematic Reviews and Meta-Analyses flow diagram and depiction of the screening and inclusion process. APK: Android Package Kit

Out of 432 apps initially identified through reseller platforms and search engines, 178 apps were included in the final analysis after duplicate removal and eligibility screening (Figure 1). Of the 178 apps, 99 apps were available on both Google Play and the App Store, 64 apps were available exclusively on Google Play, and 13 apps were exclusive to the App Store (Figure 1).

**Figure 2.**
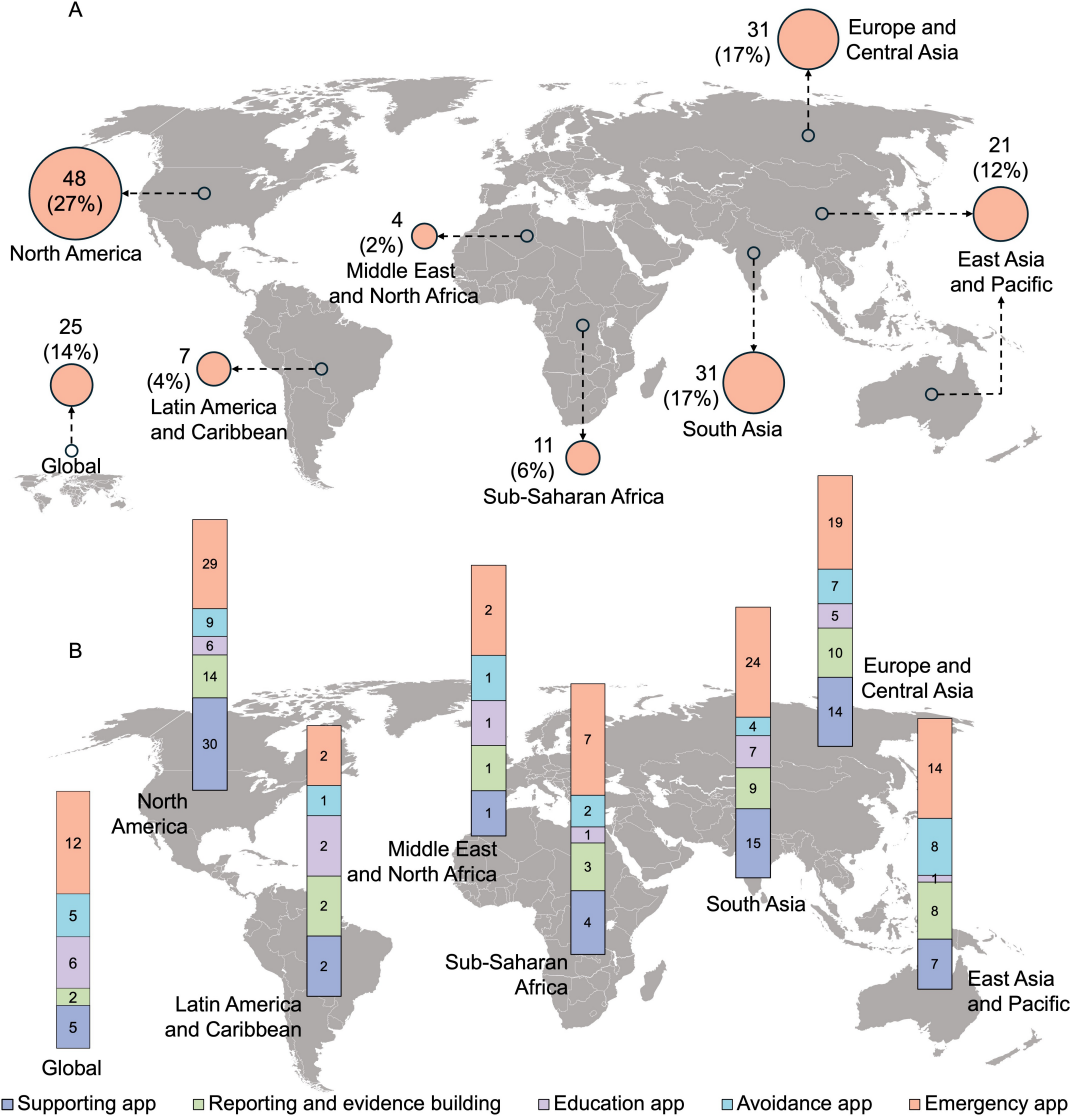
Global distribution of mobile apps: (A) Frequency and percentage by region, (B) Functional category classification across regions in percentage (multicategory classification) (N=178)

North America reported the highest implementation rate (48, 27%), with Europe and Central Asia (31, 17%), and South Asia (31, 17%) each contributing 31 apps. In North America, emergency (n=29) and support (n=30) apps were most prevalent. Similarly, in South Asia, Europe, and Central Asia, emergency (n=24 and n=19, respectively) and support apps (n=15 and n=14, respectively) were predominantly implemented (Figure 2).

### Trends of App Release

**Figure 3.**
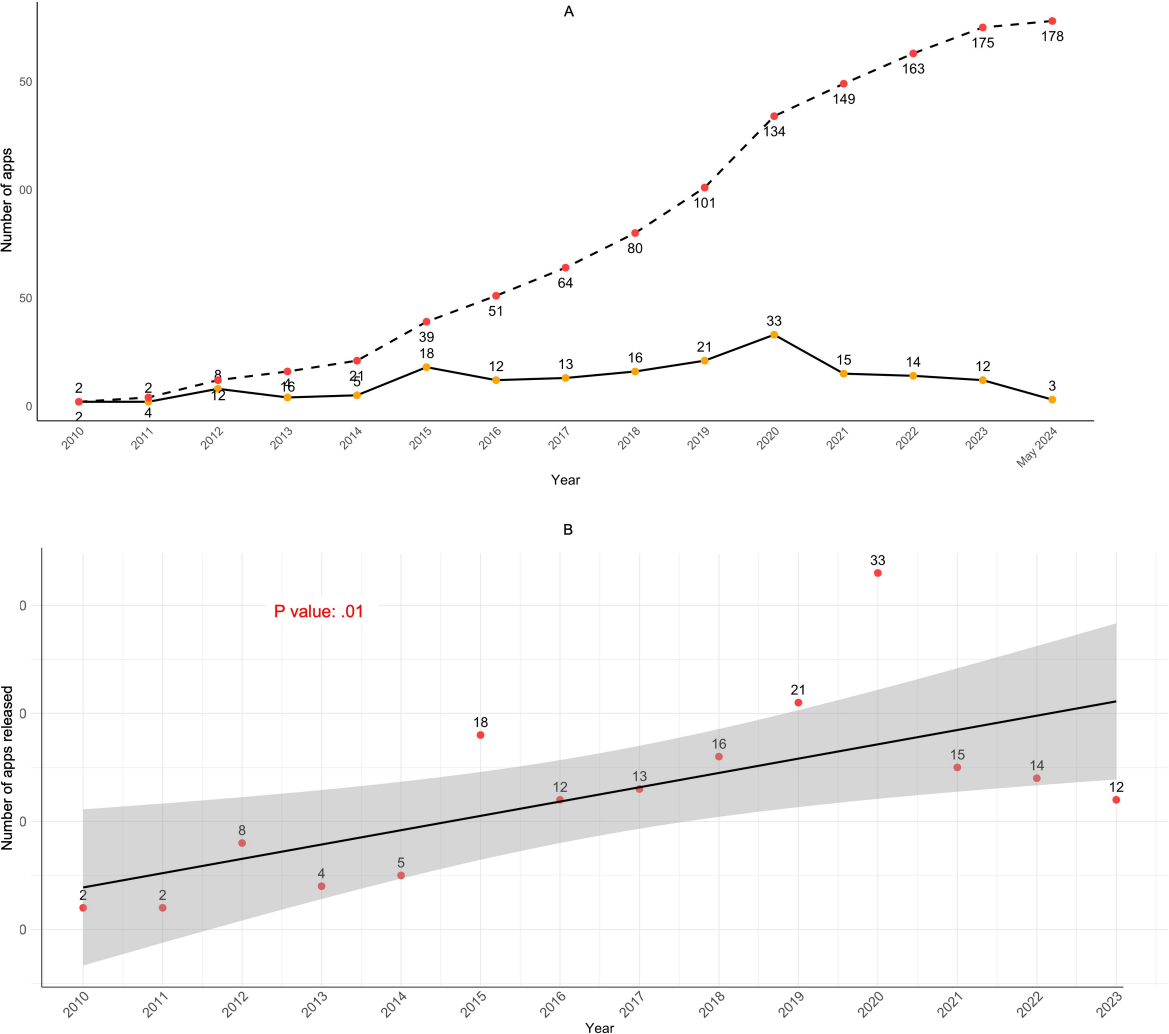
Trends in mobile app releases over time: (A) cumulative number of app releases by year and (B) annual trend in app releases with linear regression modeling (N=178).

Figure 3 illustrates the trend and cumulative number of app releases over the years and the trend analysis of app releases with regression modeling. The trend graph shows a gradual increase in app releases until reaching a peak in 2020 with 33 apps, followed by a significant decline thereafter. Besides, the cumulative graph indicates a steady overall growth from 2 to 178 between 2010 and May 2024. The regression line, with a *P*=.01, suggests a statistically significant increasing trend in app releases over the years. The shaded area represents the 95% CI for the regression line indicating a good fit for the linear model.

**Figure 4.**
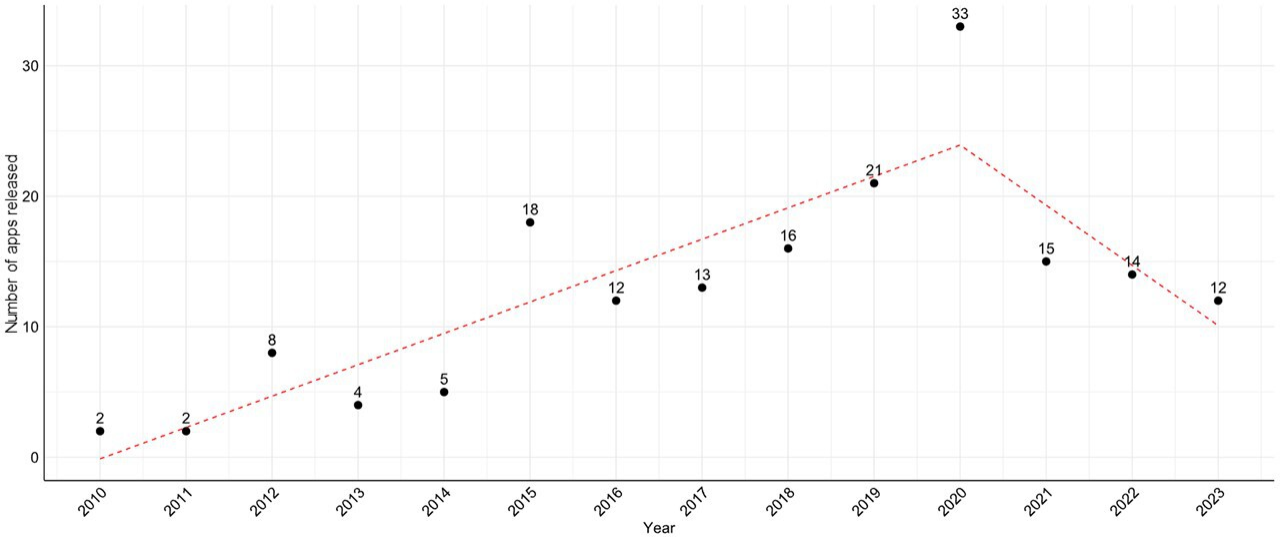
Segmented regression analysis of app releases with 2020 breakpoint (N=178).

Figure 4 demonstrates the segmented regression analysis, identifying 2020 as a breakpoint. The segmentation line suggests an initial increase in app releases until 2020, followed by a marked decline. Furthermore, the segmented regression model in Multimedia Appendix 1 presents that before the breakpoint in 2020, the number of app releases increased by approximately 2.40 units per year (*P*<.001). After 2020, the trend shifts, showing a decrease in the rate of change with a reduction of 7.01 units per year, indicating an overall decrease of 4.61 units per year in the postbreakpoint trend.

### Distribution of Apps

Table 1 presents the frequency and percent distribution of apps by sector, target groups, cost, website availability, GPS availability, and offline mode. In total, 178 apps were included in this analysis. Most apps were from the private sector (n=132, 74%), with fewer from the public sector (n=32, 18%). Most apps were designed specifically for survivors (n=121, 68%), while a smaller proportion cater to both survivors and nonsurvivors (n=57, 32%). In terms of cost, most apps were available for free without in-app purchases (n=100, 56%), while others were free but included in-app purchases (n=39, 22%). Furthermore, most apps had a website (n=148, 83%), and a considerable majority offered GPS or navigation features (n=142, 80%). However, only a small proportion provided offline functionality (n=27, 15%), while the majority did not include this feature (n=152, 85%).

Table 2 presents the frequency distribution of apps by feature type. The most common feature type was emergency and safety alerts, found in 149 apps, followed by location tracking and sharing in 90 apps and safety tips and guidance in 80 apps.

Table 3 presents the trend of prevalent features in mobile apps designed to prevent VAWG from 2010 to May 2024. Initially, the focus was on basic functions like GPS location tracking and SOS alerts (2010‐2011). Over time, features became more sophisticated, with the introduction of real-time location tracking, emergency calls, and crisis support systems (2012‐2013).

By 2014‐2016, additional safety tools like panic buttons, real-time communication, and discreet reporting were developed. From 2017 onward, apps integrated more comprehensive support services, such as educational resources, peer support, and voice activation, evolving into multifunctional platforms offering personalized safety, community engagement, and real-time emergency assistance. In recent years (2019‐2024), apps have expanded to include group communication, safe journey planning, and risk area identification.

**Table 1.** Frequency and percent distribution of apps by sector, target groups, cost, website availability, GPS availability, and offline mode (N=178).

Variable and Category		n (%)	
Sectors			
Public		32 (18)	
Private		132 (74)	
Both		14 (8)	
Target groups			
Survivors		121 (68)	
Survivors and nonsurvivors		57 (32)	
Cost			
Free without in-app purchase		100 (56)	
Free with in-app purchase		39 (22)	
Not free		35 (20)	
Unclear		4 (2)	
Website			
Yes		148 (83)	
No		30 (17)	
GPS			
Yes		142 (80)	
No		36 (20)	
Offline mode			
Yes		27 (15)	
No		151 (85)	

**Table 2.** Frequency distribution of apps by feature type (multicategory classification) (N=178).

Feature	Frequency of apps
Safety alerts	149
Location tracking and sharing	90
Safety tips and guidance	80
Communication and support	72
Reporting and documentation	54
Privacy and security measures	19
Education and awareness	16
Community engagement and collaboration	14

**Table 3.** List of app features available more than once corresponding to year of release.

Year	Prevalent features
2010	GPS location tracking SOS^a^ alerts
2011	Location information Notification services
2012	Real-time location tracker Emergency calls or alerts Contact response team Crisis support Reporting systems Create safe boundaries for added security
2013	Deliver important safety information Real-time location tracker Access to safety resources and phone numbers
2014	GPS location tracking Emergency button Emergency assistance Tracking maps, journey tracking, or mutual tracking
2015	GPS location tracking Panic buttons or send emergency and notification messages Consolidated information on specialized medical services Safe space for women Real-time communication Reporting resources Talk or chat with someone at anytime Advocacy and support services Provides steps to preserve evidence
2016	Real-time location tracker GPS location tracking Emergency button or panic button Customize messages and notifications Discreet reporting Find nearby essential services
2017	Panic buttons Report incident Live breaking video Real-time location tracker Instantly notifies police Educational resources Resource directory Comprehensive support
2018	Emergency alerts or panic button GPS location tracking Real-time location tracker Send emergency and notification messages Medical information integration Call for support Direct chat support Volunteer for social projects Learn about violence, abuse, and rights Privacy-focused design
2019	Emergency SOS alert or panic button Real-Time location tracker GPS location tracking Reporting systems Peer support program Empower users Advocacy and support services Educational resources Voice activation
2020	Instant assistance at your fingertips Verified emergency response Real-time location tracker Instant help from nearest police, hospitals, and fire In-app messaging or group chat Access to counseling for emotional support and guidance Educational resources Record incidents using multimedia 24 × 7 Toll-Free helpline
2021	Panic or emergency response button Safe location sharing Real-time location tracker Reporting system Quick alerts to emergency or trusted contacts Safe journey planner Safety or women-friendly place on map Helpline numbers Prompt dispatch of police assistance
2022	SOS feature Real-time location tracker GPS location tracking Create groups and add contacts Reporting system Risk area identification Community support or access to support
2023	SOS panic button alerts Real-time location tracker Community engagement through alerts and chats Emergency or trusted contacts Safety tips Find nearby safe places Private space for users
May 2024	Emergency alert or panic button GPS location tracking Real-time location tracker

a SOS: Save Our Souls.

### Functional Categories

**Figure 5.**
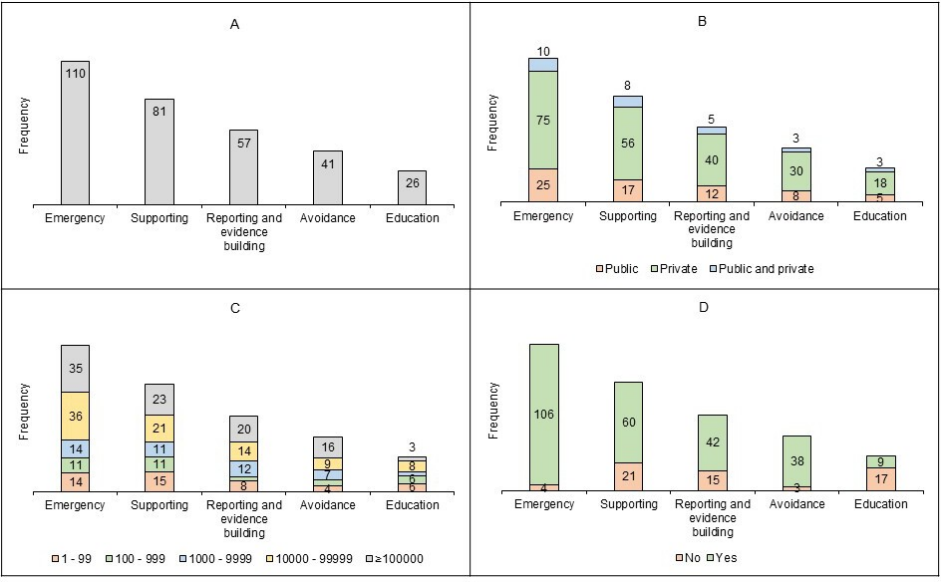
Distribution of apps by functional categories: (A) overall frequency; (B) distribution by sector; (C) distribution by number of downloads; and (D) distribution by GPS functionality (multi-category classification) (N=178).

Figure 5 presents the frequency distribution of apps by overall functional categories and the frequency distribution of functional categories of apps by sector, number of downloads, and GPS functionality. The majority of apps are categorized as an emergency (n=110), followed by supporting (n=81), and reporting and evidence building (n=57). In terms of sector distribution, most emergency apps are from the private sector (n=75), while the public sector contributes fewer apps (n=25). Similarly, supporting apps are predominantly from the private sector (n=56), with fewer from the public sector (n=17).

Regarding the number of downloads, most emergency apps fall into the 10,000 to ≥100,000 downloads category (n=71), whereas education apps have the fewest downloads in this category (n=11). For supporting apps, 44 are in the 10,000 to ≥100,000 downloads range. In terms of GPS functionality, the vast majority of emergency apps (n=106) and supporting apps (n=60) include GPS (Figure 5).

**Figure 6.**
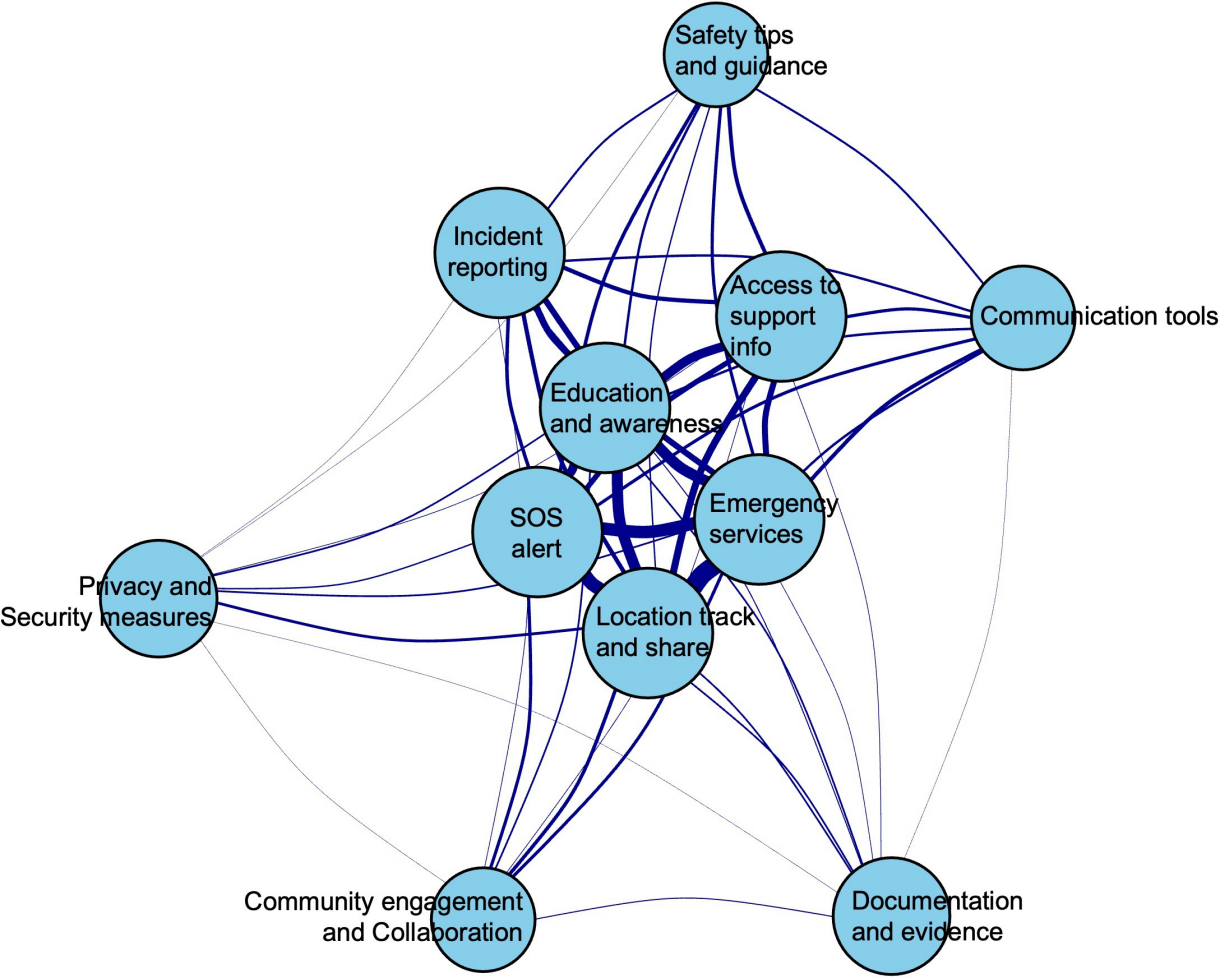
Network analysis of feature co-occurrence. SOS: save our souls.

Figure 6 presents the network analysis of feature co-occurrence. Location Track and Share, Emergency Services, Education and Awareness, and SOS Alerts have the highest degree (10) and clustering coefficient (0.911), indicating they are well-connected and form tight clusters. However, Community Engagement and Collaboration and Privacy and Security Measures stand out with higher betweenness centrality (0.585 and 0.407, respectively), serving as critical connectors. Access to Support Information has the highest closeness centrality (0.256), while Location Tracking leads in eigenvector centrality (1.0), highlighting its overall influence in the network (Figure S7 Multimedia Appendix 1).

## Discussion

### Principal Findings and Comparison With Previous Work

Our findings reveal that while the majority of apps designed to prevent VAWG predominantly focus on emergency and support functionalities, there has been a notable shift in recent trends. After an initial surge in app releases, a decline has emerged, accompanied by a growing emphasis on integrating more holistic safety solutions, including features for communication, reporting, and community engagement.

Most apps being available on both Google Play and the App Store suggests that developers recognize the importance of cross-platform compatibility to effectively reach diverse user bases. However, many apps are available exclusively on Google Play, likely due to its open nature and large user base. A similar pattern of app availability was also reported in another systematic review [6]. By not being available on multiple platforms, especially on iOS, app developers miss the opportunity to reach a broader audience, thereby limiting their market reach and potential revenue streams.

The implementation of apps across regions shows a notable concentration in North America, which accounted for the highest number of apps, particularly in emergency and support categories. This trend reflects the region’s advanced technological infrastructure and emphasis on leveraging mobile solutions for personal safety and violence prevention. North America leads in the launch and availability of apps compared to the Middle East, which may also explain the higher prevalence of mobile apps specifically aimed at preventing VAWG in this region [25]. Interestingly, a systematic review conducted in 2018 found that most apps were initially developed in South Asia [8], whereas our study focuses on the country of implementation rather than initiation. Our findings reveal that 14 apps were developed in one country and implemented in another. However, understanding the country of implementation instead of the country of development is crucial for grasping the dynamics of the need for such apps in those regions to address VAWG.

The temporal trends in app releases reveal a dynamic market response to societal needs and technological advancements. During the COVID-19 pandemic in 2020, the world experienced a considerable hike in VAWG [14] and the peak in app releases around 2020 reflects a period of intense innovation and response to increased concerns of gender-based violence issues. During the COVID-19 pandemic, digital interventions, especially for domestic and intimate partner violence, became increasingly valuable as social and physical isolation measures intensified, leaving many survivors sequestered at home with abusive partners [26]. In addition, prepandemic digital tools, such as the myPlan app (Johns Hopkins University) [27], I-DECIDE (University of Melbourne) [28], and iSafe (University of Auckland) [29], which were already recognized for their efficacy in reducing violence and providing confidential support, saw heightened importance [26]. However, the subsequent decline warrants further research into whether a maturation phase in the market, where developers may have reached a saturation point or shifted focus to refining existing solutions rather than introducing new ones. This decline may also suggest the decreased popularity of these apps, which calls for further research.

Our study shows a strong private sector presence in the development of violence prevention apps, consistent with other findings that emphasize the entrepreneurial drive to innovate in this area [8]. However, the limited involvement of public sector entities suggests a missed opportunity for leveraging these technologies within broader social and policy frameworks. Increased public sector engagement could enhance the development and dissemination of apps that are both effective and accessible to diverse populations [30].

The major focus on survivors-centered solutions, coherent with other studies [8], underscores the critical role these apps play in providing immediate safety and support, yet there remains potential for expanding features to encompass broader community engagement and preventive measures. Besides, we found a considerable majority of the apps have an associated website. A web presence can enhance credibility and offer additional resources, serving as a channel for feedback, updates, and community engagement, thus enriching the user experience.

In addition, accessibility remains a key consideration, with the majority of apps available free of charge but often requiring in-app purchases for full functionality, which is coherent with another study [8]. This model may limit access for users with financial constraints, underscoring the importance of developing inclusive strategies that ensure all users can benefit from these technologies [10]. Given that individuals of low socioeconomic status face a higher risk of violence [31], it is crucial for effective apps to be freely accessible.

Furthermore, four-fifths of the apps offer GPS or navigation features, which is a critical function for users seeking immediate help or looking to locate nearby services and resources. Another systematic review also found a similar prevalence of location-sharing features [6,9,10]. GPS functionality can significantly enhance the utility of these apps, particularly in emergency situations where users need quick access to safe locations or services. In addition, the prevalent inclusion of GPS functionality in emergency and supporting apps underscores the critical role of location-based services in increasing the effectiveness of digital interventions. By providing users with real-time assistance and access to nearby resources, GPS-enabled features align with broader research indicating their capacity to improve outcomes in crisis situations [32], thereby enhancing the overall impact and utility of these apps in violence prevention efforts. Despite these positive aspects, only 15% of the apps provide offline functionality. The lack of offline features is a significant limitation, especially in areas with unstable internet connectivity or during situations where internet access may be unavailable. Offline functionality is essential for ensuring that users can access critical information, resources, or services at any time, regardless of their connectivity status.

The landscape of functional categories of mobile apps designed to prevent VAWG is primarily populated by those categorized as emergency and support tools, indicating a societal emphasis on enhancing personal safety and empowerment through immediate digital solutions. This aligns with findings in the literature [6,8-10], which highlight the importance of digital interventions in providing real-time assistance and fostering a sense of security among users. However, contrary to another systematic review that emphasized the prominence of avoidance and education apps, our study reveals that apps can be classified into multiple functional categories, whereas the other review categorized each app into a single category [8].

Features including safety alerts, location tracking and sharing, and safety tips and guidance were prevalent, which are crucial for providing timely and effective support. The widespread inclusion of these features is coherent with other studies [10,33] and underscores the critical role of technology in empowering individuals and communities to respond swiftly to threats and seek help when needed [33]. This trend also reflects broader societal recognition of the need for accessible and reliable safety tools, as research consistently shows that users prioritize functional capabilities that enhance their sense of security and provide tangible support in crisis situations [34].

The high download numbers of emergency apps suggest widespread accessibility and potential interest, but they do not necessarily indicate significant user engagement. The considerable download numbers of the emergency apps may demonstrate a widespread popularity in digital tools that offer immediate assistance and protection. This is supported by research indicating that users are more likely to engage with apps that provide immediate intervention capabilities [35]. Nevertheless, one study found that personal safety apps may reduce fear of crime but do not necessarily decrease vulnerability to victimization, as most apps focus on intervention during or after a criminal event rather than preventing it [6]. However, educational apps, which focus on raising awareness and providing information, have a smaller user base, suggesting that users prioritize functional capabilities that directly enhance safety over purely informational content.

The network analysis reveals that features such as Location Track and Share, Emergency Services, Education & Awareness, and SOS Alerts are frequently integrated together within mobile apps, as indicated by their high degrees and clustering coefficients. This co-occurrence suggests that developers often bundle these features to create comprehensive safety solutions that address multiple aspects of user needs in a cohesive manner. In contrast, features like Community Engagement & Collaboration and Privacy & Security Measures exhibit higher betweenness centrality, serving as critical connectors that bridge different app functionalities and facilitate a seamless user experience. In addition, the prominence of Location Tracking, as indicated by its leading eigenvector centrality, underscores its overall influence and importance in the network, further highlighting developers’ focus on integrating location-tracking features to enhance app efficacy [6,9,10].

Our study also reveals a gradual shift towards more sophisticated and comprehensive safety tools, evolving from basic GPS tracking and SOS alerts to advanced features, such as real-time communication, panic buttons, peer support, and group communication, culminating in multifunctional platforms offering personalized safety, community engagement, and proactive risk identification. This evolution reflects a growing recognition of the importance of holistic approaches that address both immediate threats and long-term support, mirroring broader trends and calls [36,37].

The incorporation of advanced technologies in mobile apps aimed at preventing VAWG underscores a significant evolution in digital safety solutions. Among these innovations, the use of features like feelings trackers exemplifies the integration of nuanced and responsive technology into personal safety apps. Feelings trackers provide users with a method to monitor and record emotional changes, offering valuable insights for both immediate and long-term support. Other notable advancements include the integration of the smart pepper spray, which combines traditional self-defense mechanisms with smart technology, allowing users to trigger alerts and send their location directly from the device. The use of SOS Sirens and Flash features enhances the visibility of emergency signals, while Discreet and Disguised Interfaces provide an added layer of privacy by making the app appear as another application, thus protecting user confidentiality in sensitive scenarios. Voice Activation and Commands, along with features such as Shake Alerts and Fake Calls, contribute to the ease of use and discreet nature of the apps, allowing users to activate safety measures without drawing attention. Automatic Fall Alerts, which detect sudden impacts and send alerts to emergency contacts, further exemplify how technology can pre-emptively address critical situations.

Although United Nations Women advocates for the use of artificial intelligence (AI)-driven technologies—particularly for monitoring hate speech, fact-checking, and countering disinformation on social media, with attention to misogynistic and harmful gendered narratives [38], we found a significant lack of apps using this technology. Future applications could leverage AI and mobile phone sensors to automatically detect and respond to threats without user intervention, thereby transitioning from reactive to proactive safety measures. Such integration also aligns with recent studies emphasizing the role of AI in enhancing predictive and automated safety technologies [39]. Expanding the use of these technologies could significantly improve the effectiveness and responsiveness of safety apps, creating a more comprehensive and anticipatory safety framework.

One limitation of our study is that the search terms used may not have captured all relevant apps available on reselling platforms, as the platform’s search functionality itself may not retrieve all apps related to a given term. Besides, Google Translator was used to translate English search terms into other language, which may potentially cause errors or loss of context of the original search terms. Some apps were geo-restricted, limiting our ability to access and analyze details from outside specific countries, potentially leading to gaps in our search and analysis. We relied on diverse and broad search terms guided by previous studies, which may have introduced variability in app selection and limited the focus on apps specifically addressing violence prevention. Although this study included apps in different languages, we were unable to analyze whether language influences app features, which may be a limitation. Furthermore, we were unable to analyze app ratings and user feedback, which are critical components of app evaluation. We did not examine key metrics such as downloads, user satisfaction, user profiles, and valued features, which future research may prioritize, particularly with a focus on the pre- and post-COVID-19 impact on the use and user experience of these apps.

### Conclusion

The analysis shows that most apps prioritize emergency and support functions, with notable implementation in the United States, India, and the United Kingdom. Despite initial growth, there has been a decline in new app releases after 2020. Most apps are from the private sector, designed for survivors, and commonly offer GPS functionality and a web presence but lack offline capabilities. Features such as emergency services, location sharing, SOS alerts, and educational resources features highly co-occurred in the same app. Initially, app focused on location sharing and emergency alerts, but it expanded to include communication, reporting, incident management, community engagement, privacy protection, and comprehensive safety solutions.

Future app development should ensure cross-platform availability, including iOS, is crucial to reach a wider audience and maximize impact. Incorporating offline functionality will improve accessibility in areas with limited internet connectivity. Strengthening public sector collaboration can enhance the integration of these technologies into broader social and policy frameworks, making them more accessible. Apps should be designed inclusively, with strategies to provide free or subsidized access for users with financial constraints. In addition, integrating advanced technologies such as AI for automated threat detection and incorporating innovations like smart self-defense mechanisms will enhance the utility and responsiveness of these apps. Furthermore, a further systematic review of the literature is essential to fully understand the implementation outcomes of these apps.

## Supplementary material

10.2196/66247 Multimedia Appendix 1 Supplementary tables and figures.

## Abbreviations

AI: artificial intelligence
PRISMA: Preferred Reporting Items for Systematic Reviews and Meta-Analyses
PROSPERO: International Prospective Register of Systematic Reviews
SOS: Save our souls
VAWG: violence against women and girls

## References

[1] World Health Organisation (2024). Violence against women.

[2] Research, Trend Analysis Branch UNOOD, Crime (2021). Killings of women and girls by their intimate partner or other family members: global estimates 2020. United Nations Office on Drugs and Crime.

[3] Chrisler JC, Ferguson S (2006). Violence against women as a public health issue. Ann N Y Acad Sci.

[4] UN Women (2024). Innovation and prevention of violence against women: practice brief. United Nation Women.

[5] Storer HL, Nyerges EX, Hamby S (2022). Technology “Feels Less Threatening”: The processes by which digital technologies facilitate youths’ access to services at intimate partner violence organizations. Child Youth Serv Rev.

[6] Maxwell L, Sanders A, Skues J (2020). A content analysis of personal safety apps: are they keeping us safe or making us more vulnerable?. Violence Against Women.

[7] White D, McMillan L (2020). Innovating the problem away? A critical study of anti-rape technologies. Violence Against Women.

[8] Eisenhut K, Sauerborn E, García-Moreno C (2020). Mobile applications addressing violence against women: a systematic review. BMJ Glob Health.

[9] Draughon Moret J, Todd A, Rose L (2022). Mobile phone apps for intimate partner and sexual violence prevention and response: systematic search on app stores. JMIR Form Res.

[10] Ford K, Bellis MA, Judd N (2022). The use of mobile phone applications to enhance personal safety from interpersonal violence - an overview of available smartphone applications in the United Kingdom. BMC Public Health.

[11] Cumiskey KM, Brewster K (2012). Mobile phones or pepper spray?. Fem Media Stud.

[12] Sumra M, Asghar S, Khan KS (2023). Smartphone apps for domestic violence prevention: a systematic review. Int J Environ Res Public Health.

[13] (2024). Google play statistics and trends 2025. 42matters.

[14] Sánchez OR, Vale DB, Rodrigues L (2020). Violence against women during the COVID-19 pandemic: an integrative review. Int J Gynaecol Obstet.

[15] Doria N, Ausman C, Wilson S (2021). Women’s experiences of safety apps for sexualized violence: a narrative scoping review. BMC Public Health.

[16] Jewkes R, Dartnall E (2019). More research is needed on digital technologies in violence against women. Lancet Public Health.

[17] Page MJ, McKenzie JE, Bossuyt PM (2021). The PRISMA 2020 statement: an updated guideline for reporting systematic reviews. BMJ.

[18] (2022). WB regions and countries. World Bank Group.

[19] R Core Team R (2013). R: A language and environment for statistical computing.

[20] Wickham H (2011). ggplot2. WIREs Computational Stats.

[21] Muggeo VM (2008). Segmented: an R package to fit regression models with broken-line relationships. R news.

[22] Csardi MG (2013). Package ‘igraph.

[23] Wickham H, Averick M, Bryan J (2019). Welcome to the Tidyverse. J Open Source Softw.

[24] Gajdoš P, Ježowicz T, Uher V (2016). A parallel Fruchterman–Reingold algorithm optimized for fast visualization of large graphs and swarms of data. Swarm Evol Comput.

[25] (2023). Leading countries based on mobile app releases in 2017. Statista.

[26] Emezue C (2020). Digital or digitally delivered responses to domestic and intimate partner violence during COVID-19. JMIR Public Health Surveill.

[27] Alhusen J, Bloom T, Clough A (2015). Development of the MyPlan Safety Decision App with friends of college women in abusive dating relationships. J Technol Hum Serv.

[28] Hegarty K, Tarzia L, Valpied J (2019). An online healthy relationship tool and safety decision aid for women experiencing intimate partner violence (I-DECIDE): a randomised controlled trial. Lancet Public Health.

[29] Koziol-McLain J, Vandal AC, Wilson D (2018). Efficacy of a web-based safety decision aid for women experiencing intimate partner violence: randomized controlled trial. J Med Internet Res.

[30] Mergel I, Edelmann N, Haug N (2019). Defining digital transformation: results from expert interviews. Gov Inf Q.

[31] Waters HH, Rajkotia A, Basu Y (2004). The Economic Dimensions of Interpersonal Violence.

[32] Ranganadh A (2020). Innovations in Electrical and Electronics Engineering: Proceedings of the 4th ICIEEE 2019.

[33] Banerjee S, Maiti P, Biswas S (2024). AI Tools and Applications for Women’s Safety.

[34] Malik AS, Acharya S, Humane S (2024). Exploring the impact of security technologies on mental health: a comprehensive review. Cureus.

[35] Debnam KJ, Kumodzi T (2021). Adolescent perceptions of an interactive mobile application to respond to teen dating violence. J Interpers Violence.

[36] Emezue C, Chase JAD, Udmuangpia T (2022). Technology-based and digital interventions for intimate partner violence: a systematic review and meta-analysis. Campbell Syst Rev.

[37] (2023). Ten ways to prevent violence against women and girls. Women UN.

[38] Macau UWaUUIi (2024). Artificial Intelligence and the Women, Peace and Security Agenda in South-East Asia.

[39] Jewani VK, Ajmire PE, Chaurasia S (2024). Impact of AI on Advancing Women’s Safety: IGI Global.

