# Supplementary material for: Mobile Apps to Prevent Violence Against Women and Girls (VAWG): Systematic App Research and Content Analysis
Source: JMIR Form Res. 2025 Jun 23;9:e66247. doi: 10.2196/66247 (PMC12208507; doi:10.2196/66247)
Supplement: Multimedia Appendix 1 [file formative-v9-e66247-s001.docx]

# Multimedia Appendix

# Search terms

## Search terms for reselling platforms

1. Violence against women
2. Gender-based violence
3. Anti-gender-based violence
4. Sexual harassment
5. Women security
6. Women safety
7. Eve teasing
8. Battered women
9. Wife abuse
10. Partner abuse
11. Anti-rape
12. Interpersonal violence
13. Technology- facilitated abuse
14. Stalking
15. Psychological abuse
16. Abuse prevention
17. Survivor support
18. Violence prevention technology
19. Self-defense
20. Safety and security apps for women
21. Violence prevention strategies
22. Crisis intervention
23. Domestic abuse
24. Victim support
25. Empowerment apps for women
26. Safe environment
27. Safety alert
28. Women’s protection
29. Personal safety technology
30. Crisis management
31. Anti-violence
32. Safety tracking
33. Support for survivors of abuse
34. Sexual assault
35. Sexual violence
36. Forensic nursing
37. Rape
38. Threat
39. Emotional abuse
40. Domestic violence
41. Intimate partner violence
42. Human trafficking
43. Female safety
44. Personal safety
45. Violence prevention
46. Safety app for women
47. Harassment prevention
48. Coercive control
49. Online harassment

## Search terms for search engine database

1. Violence against women app
2. Gender-based violence app
3. Anti-gender-based violence app
4. Sexual harassment app
5. Women security app
6. Women safety app
7. Eve teasing app
8. Battered women app
9. Wife abuse app
10. Partner abuse app
11. Anti-rape app
12. Interpersonal violence app
13. Technology- facilitated abuse app
14. Stalking app
15. Psychological abuse app
16. Abuse prevention app
17. Survivor support app
18. Violence prevention technology app
19. Self-defense app
20. Safety and security apps for women
21. Violence prevention strategies app
22. Crisis intervention app
23. Domestic abuse app
24. Victim support app
25. Empowerment apps for women
26. Safe environment app
27. Safety alert app
28. Women’s protection app
29. Personal safety technology app
30. Crisis management app
31. Anti-violence app
32. Safety tracking app
33. Support for survivors of abuse app
34. Sexual assault app
35. Sexual violence app
36. Forensic nursing app
37. Rape app
38. Threat app
39. Emotional abuse app
40. Domestic violence app
41. Intimate partner violence app
42. Human trafficking app
43. Female safety app
44. Personal safety app
45. Violence prevention app
46. Safety app for women app
47. Harassment prevention app
48. Coercive control app
49. Online harassment app

# Definition of anti-VAW app categories for qualitative content analysis

| App category | Definition |
| --- | --- |
| ’Emergency app’ | This function is defined by the following eight characteristics:   - Only intended for use in emergencies. - Temporal immediacy of alert: usually right before, during, or after an incident. - Simple operation (single alarm button/shake/scream). - Sends an emergency notice to specified contacts, community workers, and/or police officers. - GPS coordinates and/or voice or video recordings may be included in the alert. - No background information about the occurrence is conveyed. - There is no information given about VAW in general. - There is no information provided about resources in situations of VAW. |
| ’Avoidance app’ | This function is defined by the following four characteristics:   - There is no direct connection between app usage and incidents. - Functions are used prior to anticipated incidents. - An avoidance approach is implemented. - It also targets forms of VAW that do not directly affect a person's physical well-being. |
| ’Education app’ | This function is defined by the following five characteristics:   - There is no clear relationship between app usage and incidents. - Functions are used prior to possible incidents. - Education is a strategy. • Health personnel are targeted. - It also targets forms of VAW that do not directly affect a person's physical well-being. |
| ‘Reporting and  evidence building app’ | This function is defined by the following seven characteristics:   - Reporting and discussing an incident are feasible even if it has passed. - Incidents can be reported during or after their occurrence. - VAW includes non-physical and non-sexual manifestations, that do not affect one's physical integrity. - Allows for contact with other users and victims. - Provides GPS information for incident mapping, which is visible to all users. - Detailed information regarding the incident, culprit, victim, and context is provided. - Promotes public awareness and encourages sharing experiences with sexual harassment and other types of violence against women. |
| ’Supporting app’ | This function is defined by the following five characteristics:   - Only use the app after a (or consecutive) VAW occurrence. - Connects users to organizations. - Informs and links to professional resources (legal, psychological, and medical). - Mostly targets types of VAW that affect the victim's physical integrity. - Frequently addressed at intimate relationship violence. |

# Feature Categorization

| Emergency and Safety Alerts | Location Tracking and Sharing | Communication and Support |
| --- | --- | --- |
| SOS Alerts   1. SOS Alerts to Trusted Contacts 2. SOS Panic Button 3. SOS Alert sends location information and audio-video 4. SOS Alert System 5. SOS Alerts in Emergencies 6. SOS of Current Location 7. Emergency SOS 8. Emergency SOS Alert 9. SOS Emergency Contacts 10. SOS Siren to get help by ringing a siren from your device 11. SOS Flash to send help signal to a distant place 12. SOS Mode to act as an emergency ad-hoc light 13. Emergency Alerts 14. Instant Distress Signal for Emergencies 15. Panic/Emergency Response Button 16. Panic Button 17. One-Press Panic Button 18. Help Button 19. Emergency Call 20. Emergency Alert 21. Request Help 22. Send Emergency Alerts Button 23. SOS Feature 24. Trigger Button 25. 3-Way Panic Alert Trigger Feature 26. Instant Alert to Personal Responders and Professional Monitoring Support   Emergency Services and Response   1. Real-Time Monitoring 2. 24/7 Emergency Help 3. 24x7 SOS Response from ADT 4. Emergency Services Directory 5. Emergency Services Contacts 6. Immediate Help 7. Verified Emergency Response 8. Emergency Facilitates 9. Receive Daily Safety Tips 10. Direct Chat Support 11. Emergency Contact Base Available 24/7 12. Support and Advice 13. Counselling and Rescue 14. Trained Team for Immediate Response 15. Progressive Web Application 16. Secure and Flexible Access 17. Direct Accessibility 18. Sense of Security Reporting 19. Reporting Tools 20. Reporting Resources 21. Report Categorized GBV Crimes 22. Instant Assistance 23. Safety Notifications   Dispatch and Assistance   1. Prompt Dispatch of Police Assistance 2. Dispatches Nearest Help to the Victim Upon Receiving SOS Alert 3. Location and Situation Details 4. Specialized Medical Services 5. Pre-Loaded Response Plans 6. Real-Time Collaboration 7. Event Documentation 8. Tracking of Service Providers' Locations (Ambulance, Police, Fire) 9. Safe Facilities 10. Quick Police Contact 11. 24x7 Toll-Free Helpline for Women in Distress | **Real-Time Location Sharing**   1. Share Real-Time Location 2. Share Locations in Real Time 3. Location Sharing with Family & Friends 4. Share Live GPS Location 5. Track and Share Real-Time Location 6. Track Loved Ones 7. Send Real-Time Location 8. Live Location Tracking 9. Geo-Located 911 10. Share Critical Location 11. Share Vital Device Information for Safety Monitoring 12. Share Location Updates 13. Sends Location Information 14. Share Audio and Video Information 15. Sends Emergency and Notification Messages 16. Emergency Message Sending 17. Share with Your Circle in a Protected Environment 18. Stay Connected with Your Circle 19. Glympse Links 20. Panic Responses Based on GPS Location 21. Integrated Safety   **GPS Tracking**   1. GPS Location Sharing 2. Location Tracking 3. Live GPS Location 4. Track Details of Abusive Behaviour 5. Track, Save, and Report 6. Progressive Web Application 7. Simple Button Push to Send Instant Message 8. Location Monitoring 9. Position & Route Sharing 10. Movement Tracking 11. Real-Time Tracking 12. History Review 13. Location Alert 14. Geolocation History 15. Instant Messaging Right in the App 16. Audio Recording 17. Live Streaming 18. Driving Awareness 19. Live Incident Feeds 20. Weather Updates 21. Nearby Facilities 22. Customizable Sharing 23. In-App Sharing 24. Web-Based Sharing 25. Customizable SOS Message 26. Geofence Alerts 27. Geofence Notifications 28. Offline GPS Tracking   **Privacy and Security**   1. Privacy-Focused Design 2. Privacy Protection 3. Secure and Flexible Access 4. User-Friendly Interface 5. Data Privacy and Security 6. Personalized Security Network 7. GPS Tracking Feature 8. Universal Use 9. Trusted Contacts Selection 10. GPS-Powered Rapid Dispatch 11. GPS Features 12. School Courses 13. Corporate Courses 14. High-Risk Profession Courses 15. Online Training Modules 16. Disability Courses 17. Chat Bot | **Communication Tools**   1. Emergency Chat 2. Group Chat 3. List of Survivors 4. Chat with Friends and Family 5. Communication 6. Communication and Logistics 7. Peer-to-Peer HelpRoom for Survivor Chat Support 8. Talk/Chat with Someone 24/7/365 9. Live Chat and Video Calling 10. Discreet Design 11. Disguised Interface 12. Direct 911 Call 13. One-Touch 9-1-1 Emergency Calling 14. Instant Notification 15. Voice Activation 16. Voice Command Activation 17. Voice Alert & Recording 18. Emergency Contacts 19. Trusted Contacts 20. Notifications 21. Additional Information About the Incident and Location   **Support and Assistance**   1. Support Directory 2. Access to Support 3. Trusted Circle Builder 4. Information Repository 5. Testimonials and Success Stories 6. Report Mechanism 7. Peer Support Program 8. Access to Support 9. Service Provider Linkage 10. Age-Specific Interventions 11. Community Support and Empowerment 12. Live Chat 13. Access Support from Trained Counsellors 14. Protection Network Information 15. Easy Help Requests 16. Locate Protection Agencies 17. Request Free Access After Ends of Free Trial 18. Every Woman Fellowship 19. Emerging Leaders Council 20. Diplomacy Days 21. Advocacy and Support Services 22. Referral Support 23. Options to Consider   **Resource and Information Access**   1. My Journal 2. Resource Directory 3. Access to Counselling, Hotlines, and Community Forums for Emotional Support and Guidance 4. Community Concern Sharing 5. Additional Advice 6. Safe Space Locator 7. Safe Stay to Find Compliant Hostels/PGs 8. Dashboard for Service Providers 9. Comprehensive Resource Information 10. Personalized Assistance Options 11. Empowerment through Knowledge 12. Resource Sharing and Information 13. National Helpline Numbers 14. Helpline Numbers Links 15. Friend Walk 16. Consolidated Information on Specialized Medical Services 17. Create Groups and Add Contacts |
| Educational and Awareness Features | **Community Engagement and Collaboration** | **Privacy and Security Measures** |
| Educational Tools   1. Educational Resources 2. Learn About Violence, Abuse, and Rights 3. Education on Different Types of Violence 4. School Courses 5. Disability Courses 6. High-Risk Profession Courses 7. Corporate Courses 8. Stakeholder Training 9. Online Training Modules 10. Self-Defense Tutorials 11. Safety Tips 12. Women Safety Tips for Providing Guidance 13. Daily Safety Tips 14. Progressive Web Application 15. Secure and Flexible Access 16. Awareness Literature 17. Voice Command Activation 18. Voice Activation 19. Voice Alert & Recording 20. Audio Recording 21. Recording Tools 22. Video Recording 23. Relaxing Visuals 24. Audio Exercises 25. Awareness Literature 26. Educational Resources 27. Learn About Violence, Abuse, and Rights 28. Engaging Stories   Information and Awareness   1. Learn About Laws Protecting Against Gender-Based Violence 2. Engage with Interactive Stories 3. Progressive Web Application 4. Secure and Flexible Access 5. Feelings Tracker to Monitor Emotional Changes 6. Women Laws 7. Safety Tips 8. Create a Safe Online Space for Children 9. Feelings Tracker to Monitor Emotional Changes 10. Resource Directory 11. Information Sharing 12. Record Experiences and Upload Multimedia 13. Record Incidents 14. Records Threats, Intimidation, or Physical Abuse 15. Risk Area Identification 16. Security Perception Reports 17. User Testimonies 18. Incident Tracking 19. Crime Reporting 20. Tip Reporting 21. Safe Reporting | **Community Involvement**   1. Community Engagement Through Alerts and Chats 2. Community Concern Sharing 3. Public Emergency and Response Services 4. Community Support and Empowerment 5. Community Engagement Through Alerts and Chats 6. Community Policing Forums 7. Resource Sharing and Information 8. Community Support and Empowerment 9. Contributes to Better Understanding of Geographical Safety Profiles 10. Community Engagement Through Alerts and Chats 11. Community Support and Empowerment 12. Community Engagement Through Alerts and Chats 13. Community Policing Forums   **Collaboration and Contribution**   1. Resource Sharing and Information 2. Volunteer Contributions and Solidarity 3. Contribution to Local Community Policing Forums 4. Social Impact Focus 5. Resource Directory 6. Testimonials and Success Stories 7. Create a Safe Online Space for Children 8. Quick Reporting 9. Incident Tracking 10. School Courses 11. Disability Courses 12. High-Risk Profession Courses 13. Stakeholder Training 14. Corporate Courses 15. Online Training Modules | **Privacy Controls**   1. Privacy Protection 2. Anonymous Reporting 3. Anonymous Usage 4. Secure PIN Protection 5. Security Measures 6. Quick Exit Button 7. Precautions Against Monitoring 8. Secure and Private 9. Disguised Interface 10. Secure and Flexible Access 11. Privacy-Focused Design 12. Privacy Protection 13. User-Friendly Interface   **Security Features**   1. Security and Safety Features 2. Security Audits 3. Anonymous Usage 4. Secure PIN Protection 5. Security Measures 6. Safe Reporting 7. Anonymous Reporting 8. Secure and Flexible Access 9. Disguised Interface 10. Voice Alert & Recording 11. Secure and Private 12. Secure PIN Protection |
| Safety Tips and Guidance | **Reporting and Documentation** |  |
| Safety Tips and Tutorials   1. Safety Tips 2. Self-Defense Tutorials 3. Women Safety Tips for Providing Guidance 4. Tips on Personal Safety, Cyber Safety, etc. 5. Receive Daily Safety Tips 6. Safety Tips 7. Real-Time Safety Alerts 8. Pre-Loaded Response Plans 9. Tips on Personal Safety, Cyber Safety, etc. 10. Practical Safety Tips for Various Situations 11. Women Safety Tips for Providing Guidance   Guidance and Recommendations   1. Provides Regular Safety Reminders 2. Safety Measures 3. Safety and Security Measures 4. Daily Reminders and Notifications 5. Online Harassment, Cyberbullying, Sexting, and Identity Theft 6. Practical Safety Tips for Various Situations 7. Resource Directory 8. Safety and Security Measures 9. Safe Space Locator 10. Safe Stay to Find Compliant Hostels/PGs 11. Safety Notifications 12. Real-Time Updates | **Incident Reporting**   1. Report a Crime 2. Incident Tracking 3. Capture Evidence 4. Evidence Collection 5. Valid Evidence 6. Incident Documentation 7. Safe Reporting 8. Report Categorized GBV Crimes 9. Incident Tracking 10. Mobile Complaints 11. Emergency Reporting 12. Sense of Security Reporting 13. Crime Reporting 14. Tip Reporting   **Documentation and Evidence**   1. Secure and Flexible Access 2. Documentation Assistance 3. Helps to Create Legally Admissible Chronological Records to Submit in Court 4. Documentation Tools 5. Audio Recording 6. Video Recording 7. Legal Resources 8. Security Audits 9. Rigorous Screening and Training for Smooth Journeys 10. Real-Time Tracking 11. Transparent Pricing 12. Payment Options |  |

# Network Analysis of App Features

| Feature | Degree | Betweenness Centrality | Closeness Centrality | Eigenvector Centrality | Clustering Coefficient |
| --- | --- | --- | --- | --- | --- |
| Location | 10 | 0.000 | 0.154 | 1.000 | 0.911 |
| Emergency | 10 | 0.000 | 0.222 | 0.997 | 0.911 |
| Education | 10 | 0.000 | 0.152 | 0.840 | 0.911 |
| SOS Alert | 10 | 0.000 | 0.164 | 0.854 | 0.911 |
| Access | 10 | 0.000 | 0.256 | 0.615 | 0.911 |
| Incident | 10 | 0.000 | 0.294 | 0.479 | 0.911 |
| Documentation | 8 | 0.007 | 0.313 | 0.245 | 1.000 |
| Safety Tips | 8 | 0.111 | 0.313 | 0.285 | 0.964 |
| Communication | 8 | 0.000 | 0.278 | 0.308 | 0.964 |
| Community | 9 | 0.585 | 0.400 | 0.168 | 0.944 |
| Privacy | 9 | 0.407 | 0.370 | 0.141 | 0.944 |

# Segmented regression model results by analysis of temporal trends with identified breakpoint in 2020.

| Parameter | Estimate | Standard Error | t Value | p-Value | Multiple R-Squared | Adjusted R-squared |
| --- | --- | --- | --- | --- | --- | --- |
| Intercept | -4832.48 | 1032.45 | -4.68 | 0.0009 *** | 0.7628 | 0.6916 |
| Year | 2.40 | 0.51 | 4.69 | 0.0009 *** |  |  |
| U1.Year | -7.01 | 2.14 | -3.27 | - |  |  |
| Estimated Breakpoint | 2020 | 0.72 | - | - |  |  |

*Note: Significance codes: ***p < 0.001, **p < 0.01, *p < 0.05*

# Word frequency/crowding analysis

| **Word** | **Frequency** |
| --- | --- |
| Location | 95 |
| Emergency | 89 |
| Report | 49 |
| Information | 48 |
| Safety | 46 |
| Help | 44 |
| Support | 39 |
| Services | 39 |
| Alerts | 39 |
| Contact | 37 |
| Real-time | 35 |
| SOS | 34 |
| GPS | 34 |
| Access | 34 |
| Notification | 32 |
| Tracking | 32 |
| Sharing | 31 |
| Share | 30 |
| Reporting | 28 |
| Panic | 27 |
| Call | 25 |
| Resources | 21 |
| Response | 22 |
| Chat | 20 |
| Assistance | 20 |
| Police | 20 |
| Violence | 16 |
| Instant | 16 |
| Live | 14 |
| Family | 13 |
| Incident | 12 |
| Immediate | 12 |
| Monitoring | 12 |
| Security | 11 |
| Tracker | 11 |
| Community | 10 |
| Voice | 10 |
| Quick | 10 |
| Women | 9 |
| Secure | 9 |
| Anonymous | 9 |
| Battery | 8 |
| Specialized | 8 |
| Child | 8 |
| Plan | 8 |
| Prevent | 7 |
| Education | 7 |
| Advocacy | 7 |
| Training | 7 |
| Privacy | 7 |
| Create | 7 |
| Comprehensive | 7 |
| Online | 7 |
| Children | 7 |
| Local | 7 |
| Crime | 7 |
| Phone | 7 |
| Discreet | 7 |
| Communication | 7 |
| Immediate Help | 6 |
| Domestic | 6 |
| Check-in | 6 |
| Control | 6 |
| Reports | 5 |
| Consolidated | 5 |
| Professional | 5 |
| Prevention | 5 |
| User-friendly | 5 |
| Directory | 5 |
| Device | 5 |
| Group | 5 |
| Network | 5 |
| Protection | 5 |
| Awareness | 4 |
| Direct | 4 |
| Remote | 4 |
| Management | 4 |
| System | 4 |
| Private | 4 |
| Public | 4 |
| Health | 4 |
| Peer | 4 |
| Customizable | 3 |
| Personalized | 3 |
| Volunteer | 3 |
| Simple | 3 |
| Location-based | 3 |
| Fall | 3 |
| Mobile | 3 |
| Law | 3 |
| Connection | 3 |
| Arrival | 2 |
| Kids GPS Tracker | 2 |
| Event | 2 |
| Framework | 2 |
| Locator | 2 |
| Emergency Reporting | 2 |
| Accompaniment | 2 |
| Compliance | 2 |
| Solidarity | 2 |
| Violations | 2 |
| Tool | 2 |
| High-Risk | 2 |
| Hazard | 2 |
| Visual | 2 |
| Coordination | 2 |
| Hotline | 1 |
| Confidential | 1 |
| Dangerous | 1 |
| Crowdsource | 1 |
| Recovery | 1 |
| Certified | 1 |
| Forensic | 1 |
| Follow-up | 1 |
| Story | 1 |
| Criminal | 1 |
| Family GPS Tracker | 1 |
| Retrieval | 1 |
| Illegal | 1 |
| Fraud | 1 |
| Preserving Evidence | 1 |
| Collaboration | 1 |
| Non-Movement | 1 |
| Holistic | 1 |
| Legal Information | 1 |
| Screening | 1 |
| Custom | 1 |
| School Violence | 1 |
| Digital | 1 |
| Testimonies | 1 |
| Map Visual | 1 |
| Geo-Location | 1 |
| Navigation | 1 |
| Protected | 1 |

# List of all features

1. Includes location and Google Maps link
2. Share real-time location
3. Real-time via GPS
4. Share location
5. Live GPS
6. Send real-time location
7. Real-Time Monitoring
8. Track and share real-time location
9. Real-time GPS location
10. Share location updates
11. Panic responses based on GPS location
12. GPS tracking
13. Child Location Tracking
14. Remote Location Tracking
15. GPS Location Tracking
16. Share live GPS location
17. Share critical location
18. Monitor real-time location
19. Safe Location Sharing
20. Location Sharing in Threat
21. Location Sharing with Trusted Contacts
22. Continuous Real-Time Location Sharing
23. Geographical Coverage
24. Automatic Location Updates
25. Geolocation tracking
26. Location-based Services
27. Latitude, Longitude, Street address
28. Geo-Location Sharing
29. Incident Tracking
30. Real-Time Journey Sharing
31. Emergency Contact Notification
32. Sends email to preconfigured email ID(s)
33. Emergency triggers
34. Trigger alarm
35. 24/7 emergency help
36. Real-time alerts
37. Emergency contact base available 24/7
38. Send alerts via text messages, push notifications, and emails
39. Call 911, emergency responders
40. Send alerts to trusted contacts
41. Prioritize immediate attention
42. Automatic forwarding of detailed emergency calls
43. Send message with location
44. Emergency response
45. 24/7 efficient response involving local volunteers
46. Alerts via SMS if GPRS is unavailable
47. Real-Time Alerts
48. 24x7 Call center assistance in emergencies
49. Emergency Calls/Alerts
50. Direct 911 Call
51. Send messages to trusted friends
52. Customizable Alert Message
53. Instant distress signal for emergencies
54. Emergency Help and Support Numbers
55. Emergency Alerts and Communication
56. Emergency Notifications
57. Instant Assistance at Your Fingertips
58. Emergency Contacts
59. Instant messaging right in the app
60. Emergency Signal
61. Shows safest and most dangerous areas in the city.
62. Offers tips on personal safety, cyber safety, etc.
63. Safety Checks
64. Safe journey planner
65. Receive daily safety tips
66. Awareness literature
67. Safe journey planner
68. Deliver important safety information
69. Receive safety information
70. View safety news and alerts
71. Convenient access update
72. Training on Domestic Violence Prevention
73. Intuitive, interactive, and educational exposure
74. Details intimate partner abuse and reporting options
75. Family Advocacy Program
76. Status updates on the way to Office/Home
77. Contributes to better understanding of geographical safety profiles
78. Informs community safety activities
79. Safety Tips
80. Self-Defense
81. My Journal
82. Safety Assessment
83. Educational Resources
84. Additional Advice
85. Emergency SOS Alert
86. Voice Activation
87. Emergency SOS
88. Emergency SOS: Press crown five times
89. SOS panic button alerts
90. Panic / emergency response button
91. Silently call f with phone shake or screen button.
92. One-press panic button
93. Emergency call button
94. Send photo with location
95. Send audio & video with location
96. PANIC Button press
97. Voice Recognition
98. Volume Button Toggle
99. SOS Alert
100. Sidekick Panic Button
101. SOS Mode to act as an emergency ad-hoc light.
102. SOS Siren to get help by ringing a siren from your device.
103. SOS Flash to send help signal to a distant place.
104. Shake for Emergency
105. Double-Click Activation
106. Trigger Button
107. Voice activation
108. Hands-Free Activation
109. Manual SOS
110. Emergency SOS button
111. SOS Widget
112. SOS Alert Button
113. Help Button
114. Press the ‘SaveME 999 POLIS’ icon
115. Find Support
116. Access support from trained counselors.
117. Quick Access Help Widget
118. Support Directory
119. Online Counseling with Certified Counselors
120. Provides steps to preserve evidence post-assault.
121. Advocacy and Support Services
122. Access NGO and counselor advice
123. Specific information about resources for survivors of sexual assault
124. Remote Support
125. Support for Domestic Violence Victims
126. Professional Support and Services
127. Volunteer Contributions and Solidarity
128. Social Worker Assistance
129. Support and Advice
130. Payment Options
131. Nighttime Support Finder
132. Access to counseling, hotlines, and community forums for emotional support and guidance.
133. Quality and holistic care for survivors and communities.
134. Seek or provide anonymous advice from professional counselors and peers.
135. Peer Support Program
136. Crisis Support
137. List of Survivors
138. Counseling, and rescue
139. Online Counseling with Certified Counselors
140. Access NGO and counselor advice
141. Volunteer for social projects
142. support specialists by 24/7
143. Financial Support for Survivors
144. Helps to create legally admissible chronological records to submit in court.
145. Documentation and Reports
146. Safe Reporting
147. Activity Monitoring and Reporting
148. Anonymous Reporting
149. Mobile Complaints
150. Report a crime
151. Quick reporting
152. Report crime
153. Report lost or stolen articles
154. Track, save and report
155. Report misconduct
156. Community Incident Reporting
157. Anonymously Report Incidents
158. Comprehensive Reporting
159. Report anonymously
160. Confidential reporting
161. Report Mechanism
162. Incident Reporting
163. Risk and Hazard Reporting
164. Report Incident
165. Tip Reporting
166. Offline Reporting
167. Citizen Police to report:
168. Report Categorized GBV Crimes
169. Reporting Tools
170. Emergency Reporting
171. Discreet Reporting
172. Emergency Reporting
173. Crowdsourced Hazard Reporting
174. Report function for missing children
175. Reporting related to sexual violence, school violence, and domestic violence.
176. Security Perception Reports
177. Street Harassment Reports
178. Reporting Resources
179. Health and Safety Reporting
180. Captures two pictures (front and back camera).
181. Captures either a video or an audio clip.
182. Uploads captured media to a server.
183. Sends email to preconfigured email ID(s) with links to uploaded media.
184. Live Streaming
185. Automatic Audio and Video Recording
186. SOS alert sends location information and audio-video
187. Live Chat and Video Calling
188. Surveillance Camera Coverage
189. Syncs recordings
190. Records threats, intimidation, or physical abuse
191. Share audio and video information
192. Live Breaking Video
193. Record of audio and video evidence
194. Voice Alert & Recording
195. Self-Care: Mood Tracker, Relaxing Visuals, Audio Exercises.
196. Capture Evidence
197. Voice Recording
198. Send pictures and videos
199. Record experiences and upload multimedia
200. Audio Recording
201. eBodyCam Feature
202. Medical Info and Emergency Contacts
203. Get other people's experience, and medical advice.
204. Save and share pertinent personal medical information
205. Specialized Medical Services
206. Access information on support services and contact details for police and health facilities
207. Specialized Medical Services (Sexual Assault and Relationship Violence Treatment Forensic Exams, Washington Hospital Center, Follow Up Services)
208. Tracking of Service Providers' locations (Ambulance, Police, Fire)
209. ASL Resources for the Deaf and Hard-of-Hearing
210. Medical Information Integration
211. First Aid Assistance
212. Mental Health Support
213. Women's safety for distressed women
214. Emergency Medical Services
215. Blood Donation
216. Stay connected with your circle.
217. Chat with friends and family.
218. Create groups and add contacts
219. Real-time Chat
220. Share with your circle in a protected environment.
221. Group creation for tracking purposes
222. Community engagement through alerts and chats
223. Emergency Chat
224. Group Chat
225. Direct Chat Support
226. Talk/chat with someone 24 hours a day
227. Family chat
228. Trusted Circle Builder
229. Chat Bot
230. Join larger community
231. Private and Group Chat
232. Talk/Chat with Someone 24/7/365
233. Utilize the Beacon
234. Offline GPS tracking
235. Set browsing to kid-friendly websites
236. User-Friendly Interface for Students
237. Order AI to get immediate access
238. Bluetooth Low Energy Connectivity
239. Web-Based Sharing
240. Web-based Version
241. Integration with Android Wearable Devices
242. Activate an ear-piercing volume sound
243. Wear OS Support
244. Advanced user-friendly search
245. User-Friendly Interface
246. Works offline in a remote location.
247. Progressive Web application
248. SABRE SMART Pepper Spray Integration
249. Fake Calls
250. Anonymous Usage
251. Secure PIN Protection
252. Quickly exit screens with important information for enhanced privacy.
253. Securely search internet
254. Private space for users
255. Secure Data Encryption
256. Anonymous Usage
257. Data privacy and security
258. Quick Exit Button
259. Anonymous Access
260. Verified User Registration
261. Security PIN
262. Privacy Control
263. Automatic Fall Alerts

Secure and Private Add Code

# List of all apps according to the number of downloads

| New Sl | app name | **Region of implementation** | **Year of release** | **Year of last update** | **Number of downloads** | **Overall ratings** | **Availability on reselling platform(s) (app Store, Google Play, Windows Store or both)** | **Emergency app, Reporting and evidence building app** |
| --- | --- | --- | --- | --- | --- | --- | --- | --- |
| 1 | Life360 | North America | 2010 | 2024 | >100,000,000 | 4.6 | google play, app store | Emergency app, Supporting app |
| 2 | FlashGet Kids: parental control | North America | 2015 | 2024 | >50,000,000 | 4.7 | google play, app store | Avoidance app, Supporting app |
| 3 | Find my kids: Location Tracker | Global | 2015 | 2024 | >50,000,000 | 4.5 | google play, app store | Avoidance app, Emergencey app, Reporting and evidence building app |
| 4 | Phone Tracker and GPS Location | Europe & Central Asia | 2023 | 2024 | >50,000,000 | 4.1 | google play | Avoidance app |
| 5 | iSharing | Global | 2012 | 2024 | >10,000,000 | 4.6 | google play, app store | Emergency app, Reporting and evidence building app |
| 6 | Find my Phone - Family Locator | Global | 2013 | 2024 | >10,000,000 | 4.5 | google play, app store | Reporting and evidence building app |
| 7 | Pingo by Findmykids | Global | 2018 | 2024 | >10,000,000 | 4.6 | google play, app store | Emergency app, Reporting and evidence building app |
| 8 | MobilePatrol Public Safety app | Sub-Saharan Africa | 2013 | 2023 | >5,000,000 | unclear | google play, app store | Avoidance app, Reporting and evidence building app |
| 9 | Guardians from Truecaller | South Asia | 2021 | 2023 | >5,000,000 | 4.5 | google play, app store | Emergency app, Reporting and evidence building app |
| 10 | Parental Control app- FamiSafe | East Asia & Pacific | 2018 | 2024 | >5,000,000 | 4.2 | google play, app store | Supporting app |
| 11 | Disha SOS | South Asia | 2020 | 2024 | >5,000,000 | unclear | google play | Emergency app, Supporting app |
| 12 | Glympse - Share GPS location | Global | 2020 | 2024 | >5,000,000 | 4.4 | google play, app store | Avoidance app |
| 13 | bSafe - Never Walk Alone | Global | 2011 | 2024 | >1,000,000 | 4.2 | google play | Emergency app, Reporting and evidence building app, Supporting app |
| 14 | Noonlight: Feel Protected 24/7 | North America | 2018 | 2024 | >1,000,000 | 4.4 | google play | Supporting app |
| 15 | 112 India | Europe & Central Asia | 2018 | 2023 | >1,000,000 | unclear | google play | Emergency app |
| 16 | Emergency+ | East Asia & Pacific | 2020 | 2023 | >1,000,000 | unclear | google play, app store | Emergency app |
| 17 | SHEROES: Learn Earn Community | South Asia | 2015 | 2023 | >1,000,000 | 4.6 | google play | Supporting app |
| 18 | Citizen: Local Safety Alerts | North America | 2017 | 2024 | >1,000,000 | unclear | google play, app store | Avoidance app, Reporting and evidence building app |
| 19 | SoSecure by ADT: Safety app | North America | 2021 | 2024 | >1,000,000 | unclear | google play, app store | Emergency app, Supporting app |
| 20 | Microsoft Family Safety | Global | 2020 | 2024 | >1,000,000 | 4.4 | google play, app store, microsoft store | Supporting app |
| 21 | Family360 - GPS Live Locator | South Asia | 2021 | 2024 | >1,000,000 | 4.8 | google play | Supporting app |
| 22 | FamiOn: GPS Location Tracker | Global | 2023 | 2024 | >1,000,000 | 4.6 | google play | Avoidance app, Emergencey app, |
| 23 | Reach Out Editions | North America | 2016 | 2021 | >1,000,000 | 4.6 | google play, app store | Supporting app |
| 24 | 안전Dream - 아동·여성·장애인경찰지원센터 (Safety Dream - Police Support Center for Children, Women, and the Disabled) | East Asia & Pacific | 2011 | 2024 | >1,000,000 | unclear | google play | Emergency app, Reporting and evidence building app |
| 25 | AirDroid Parental Control | East Asia & Pacific | 2022 | 2024 | >1,000,000 | 4.7 | google play, app store | Avoidance app, Supporting app |
| 26 | Connected: Locate Your Family | Europe & Central Asia | 2020 | 2024 | >1,000,000 | 4.5 | google play, app store | Emergency app, Avoidance app, Supporting app |
| 27 | "Hawk Eye - Telangana Police" | South Asia | 2015 | 2021 | >500,000 | unclear | app store | Emergency app, Reporting and evidence building app |
| 28 | Zenly Share Location - Penlo | East Asia & Pacific | 2022 | 2023 | >500,000 | 4.6 | google play, app store | Avoidance app |
| 29 | CitizenCOP | North America | 2012 | 2024 | >500,000 | unclear | google play, app store | Emergency app, Reporting and evidence building app |
| 30 | Namola | Sub-Saharan Africa | 2017 | 2024 | >500,000 | unclear | google play | Emergency app |
| 31 | AlertCops | Europe & Central Asia | 2018 | 2023 | >500,000 | unclear | google play, app store | Emergency app |
| 32 | DoD Safe Helpline | North America | 2012 | 2024 | >500,000 | 3.9 | google play, app store | Education app, Supporting app |
| 33 | LiveSafe | North America | 2013 | 2024 | >500,000 | 2.9 | google play | Emergency app, Reporting and evidence building app, Supporting app |
| 34 | DigiPolice | East Asia & Pacific | 2018 | 2024 | >237,000 | unclear | google play, app store | Avoidance app, Reporting and evidence building app |
| 35 | Women Safety | North America | 2015 | 2021 | >100,000 | 4.4 | google play | Education app |
| 36 | Punjab Police-Women Safety app | North America | 2021 | 2024 | >100,000 | 4.5 | google play | Emergency app |
| 37 | Nirbhayam | East Asia & Pacific | 2020 | 2021 | >100,000 | unclear | google play, app store | Emergency app |
| 38 | Smart 24×7 | South Asia | 2020 | 2024 | >100,000 | 4.1 | google play | Emergency app |
| 39 | Himmat Plus | South Asia | 2018 | 2020 | >100,000 | 4.4 | google play | Emergency app |
| 40 | Talking ParentsTalkingParents: Co-Parent app | North America | 2016 | 2024 | >100,000 | unclear | google play, app store | Supporting app |
| 41 | Our Family Wizard | North America | 2012 | 2024 | >100,000 | 4.5 | google play, app store | Supporting app |
| 42 | Red Panic Button | Europe & Central Asia | 2010 | 2023 | >100,000 | 3.6 | google play, app store | Emergency app |
| 43 | Kaaval Uthavi | South Asia | 2020 | 2023 | >100,000 | unclear | google play, app store | Emergency app, Reporting and evidence building app, Supporting app |
| 44 | Hollie Guard - Personal Safety | Europe & Central Asia | 2015 | 2024 | >100,000 | unclear | google play, app store | Emergency app, Reporting and evidence building app |
| 45 | SafeZone | East Asia & Pacific | 2012 | 2024 | >100,000 | unclear | google play, app store | Emergency app, Supporting app |
| 46 | SOS Alert \| Panic Button | North America | 2020 | 2024 | >100,000 | 4.8 | google play | Emergency app |
| 47 | Abhivyakti - Women Safety app | South Asia | 2023 | 2024 | >100,000 | unclear | google play, app store | Emergency app, Education app |
| 48 | 181 Abhayam Women Helpline | South Asia | 2020 | 2023 | >100,000 | unclear | google play, app store | Reporting and evidence building app, Supporting app |
| 49 | Rev Voice Recorder and Memos | North America | 2020 | 2024 | >100,000 | 4.3 | app store | Emergency app, Reporting and evidence building app, Supporting app |
| 50 | STOPit | Global | 2017 | 2024 | >100,000 | unclear | google play, app store | Reporting and evidence building app |
| 51 | The SPOT app | Global | 2014 | 2023 | >100,000 | unclear | google play, app store | Avoidance app |
| 52 | SOS Alert \| Emergency & Safety | South Asia | 2021 | 2024 | >100,000 | 4.7 | google play | Emergency app |
| 53 | mySOS SA | Sub-Saharan Africa | 2014 | 2024 | >100,000 | unclear | google play, app store | Emergency app, Supporting app |
| 54 | PSCA - Public Safety | South Asia | 2021 | 2023 | >100,000 | 4 | google play, app store | Emergency app, Avoidance app |
| 55 | 144 | Latin America & Caribbean | 2018 | 2023 | >100,000 | unclear | google play, app store | Supporting app |
| 56 | SOSFem - Panic/SOS Button | Latin America & Caribbean | 2019 | 2024 | >100,000 | 4.5 | google play, app store | Emergency app |
| 57 | THE SORORITY | Global | 2022 | 2024 | >100,000 | 5 | google play, app store | Supporting app, Avoidance app |
| 58 | Ansimi | East Asia & Pacific | 2018 | 2024 | >100,000 | unclear | google play | Emergency app, Avoidance app, Supporting app |
| 59 | UrSafe: Safety & Security app | Global | 2019 | 2024 | >50,000 | unclear | google play | Emergency app |
| 60 | My Safetipin | South Asia | 2016 | 2024 | >50,000 | unclear | google play | Emergency app, Supporting app |
| 61 | Donna: Safe women - SOS | East Asia & Pacific | 2023 | 2024 | >50,000 | 4.7 | google play | Emergency app |
| 62 | SOS.Mobile | North America | 2016 | 2024 | >50,000 | 4.6 | google play, app store | Emergency app |
| 63 | BrightSky | Europe & Central Asia | 2019 | 2024 | >50,000 | 3.3 | google play, app store | Supporting app, Reporting and evidence building app, Education app |
| 64 | Aspire News app | North America | 2019 | 2022 | >50,000 | 3.4 | google play, app store | Emergency app |
| 65 | app-Elles | Europe & Central Asia | 2015 | 2024 | >50,000 | 4.1 | google play, app store | Emergency app, Supporting app |
| 66 | Kinetic Global | North America | 2012 | 2023 | >50,000 | 4.4 | google play, app store | Emergency app, Reporting and evidence building app |
| 67 | Grannus - PHPN app | South Asia | 2018 | 2024 | >50,000 | unclear | google play | Supporting app, Emergency app |
| 68 | Riding Pink Passenger | East Asia & Pacific | 2019 | 2023 | >50,000 | unclear | google play, app store | Emergency app |
| 69 | app-Elles | Europe & Central Asia | 2015 | 2024 | >50,000 | unclear | google play, app store | Emergency app, Supporting app |
| 70 | Mujeres Seguras | Latin America & Caribbean | 2019 | 2024 | >50,000 | 4.9 | google play, app store | Emergency app |
| 71 | UMAY | Global | 2019 | 2024 | >50,000 | unclear | google play, app store | Emergency app, Avoidance app, Supporting app |
| 72 | KommGutHeim – Standort teilen | Global | 2014 | 2023 | >50,000 | 4.5 | google play, app store | Emergency app, Avoidance app |
| 73 | SaveME 999 POLIS | East Asia & Pacific | 2019 | 2023 | >50,000 | unclear | google play | Emergency app, Avoidance app |
| 74 | I'm Safe - Women Safety app | Global | 2022 | 2024 | >10,000 | 4 | google play | Reporting and evidence building app |
| 75 | SHESafe | Europe & Central Asia | 2020 | 2023 | >10,000 | unclear | google play | Education app, Emergency app, Supporting app |
| 76 | Safety app for Silent Beacon | Europe & Central Asia | 2016 | 2024 | >10,000 | 5 | google play | Emergency app |
| 77 | Domestic Violence Prevention | North America | 2017 | 2022 | >10,000 | 2.9 | google play | Education app |
| 78 | Raksha — Women Safety Alert | South Asia | 2020 | 2024 | >10,000 | unclear | google play | Emergency app, Education app |
| 79 | GetHomeSafe - Personal Safety | East Asia & Pacific | 2014 | 2024 | >10,000 | unclear | google play, app store | Emergency app, Reporting and evidence building app |
| 80 | SpeakUp | South Asia | 2021 | 2022 | >10,000 | 4.6 | google play | Emergency app |
| 81 | SOSapp : Emergency SOS app | South Asia | 2019 | 2022 | >10,000 | unclear | google play | Emergency app |
| 82 | Iyzil- Your Fearless Companion | South Asia | 2021 | 2024 | >10,000 | unclear | google play, app store | Emergency app, Supporting app |
| 83 | EraStop - AgreStop | Europe & Central Asia | 2017 | 2023 | >10,000 | unclear | google play, app store | Emergency app, Supporting app |
| 84 | Safe365 | Europe & Central Asia | 2019 | 2023 | >10,000 | 4.1 | google play, app store | Reporting and evidence building app |
| 85 | StaySafe Lone Worker | Europe & Central Asia | 2020 | 2024 | >10,000 | 3 | google play, app store | Emergency app |
| 86 | SafeTTC | North America | 2017 | 2024 | >10,000 | 4.3 | google play, app store | Reporting and evidence building app |
| 87 | SHAKTI | South Asia | 2017 | 2023 | >10,000 | unclear | google play | Emergency app, Supporting app |
| 88 | Ellas | North America | 2020 | 2023 | >10,000 | unclear | google play, app store | Education app, Supporting app |
| 89 | Vive Segura CDMX | Latin America & Caribbean | 2015 | 2016 | >10,000 | unclear | google play | Avoidance app, Reporting and evidence building app, and Education app |
| 90 | Alerte 3117 | Europe & Central Asia | 2016 | 2023 | >10,000 | unclear | google play, app store | Reporting and evidence building app |
| 91 | Women's Network | Latin America & Caribbean | 2020 | 2023 | >10,000 | unclear | google play | Supporting app |
| 92 | Domestic Violence Act 2005 | South Asia | 2015 | 2024 | >10,000 | unclear | google play | Education app |
| 93 | ほくとポリス (Hokuto Police) | East Asia & Pacific | 2022 | 2023 | >10,000 | unclear | google play | Emergency app, Avoidance app, Reporting and evidence building app |
| 94 | Child Safety app | South Asia | 2020 | 2024 | >10,000 | unclear | google play | Emergency app, Supporting app |
| 95 | GuardMe 2.0 | Sub-Saharan Africa | 2020 | 2024 | >10,000 | 4 | google play, app store | Emergency app |
| 96 | SafetyLine | North America | 2020 | 2024 | >10,000 | unclear | google play, app store, microsoft store | Supporting app |
| 97 | Help Me | East Asia & Pacific | 2012 | 2019 | >10000 | 4.4 | app store | Emergency app, Supporting app |
| 98 |  |  |  |  |  |  |  |  |
| 99 | Safe And The City | Europe & Central Asia | 2023 | 2024 | >5,000 | 3.9 | google play, app store | Emergency app, Reporting and evidence building app, Supporting app |
| 100 | Daisy | East Asia & Pacific | 2015 | 2024 | >5,000 | 2.9 | google play, app store | Supporting app, Reporting app and evidence building app |
| 101 | Sexual Harassment of Women Act | South Asia | 2015 | 2024 | >5,000 | unclear | google play | Education app |
| 102 | WanderSafe Safety app | North America | 2019 | 2024 | >5,000 | 3.8 | google play, app store | Emergency app, Avoidance app, Reporting and evidence building app |
| 103 | RAINN | North America | 2020 | 2024 | >5,000 | 4.1 | google play, app store | Supporting app, Education app |
| 104 | Satark India -Women Safety app | South Asia | 2022 | 2023 | >5,000 | unclear | google play, app store | Emergency app, Avoidance app |
| 105 | SafePal | Sub-Saharan Africa | 2018 | 2023 | >5,000 | unclear | google play, app store | Reporting and evidence building app, Supporting app |
| 106 | Safelet – Safety Bracelet | Europe & Central Asia | 2016 | 2024 | >5,000 | unclear | google play, app store | Emergency app, Avoidance app |
| 107 | Panic Button With Voice Comman | Europe & Central Asia | 2019 | 2019 | >5,000 | unclear | google play, app store | Emergency app |
| 108 | Nokaneng (Lesotho) | Global | 2018 | 2024 | >5,000 | unclear | google play | Supporting app, Reporting and evidence building app |
| 109 | WalkSafe+ | Europe & Central Asia | 2023 | 2024 | >5,000 | 4.8 | google play, app store | Emergency app, Avoidance app, Supporting app |
| 110 | Walk me home - WMH | Europe & Central Asia | 2017 | 2023 | >5,000 | unclear | google play | Emergency app |
| 111 | Empower You | East Asia & Pacific | 2020 | 2023 | >3,000 | 4.2 | google play, app store | Emergency app, Supporting app |
| 112 | Círculo | North America | 2021 | 2023 | >1,000 | 3 | google play, app store | Emergency app, Supporting app |
| 113 | Street safe | Europe & Central Asia | 2022 | 2023 | >1,000 | 2 | google play | Reporting and evidence building app |
| 114 | eBodyGuard | North America | 2019 | 2024 | >1,000 | 4.7 | google play, app store | Emergency app |
| 115 | myPlan | Global | 2022 | 2024 | >1,000 | 4.2 | google play, app store | Education app, Supporting app |
| 116 | Sunny app | East Asia & Pacific | 2018 | 2024 | >1,000 | 4.2 | google play, app store | Education app, Emergency app, Supporting app |
| 117 | Tozi | Europe & Central Asia | 2023 | 2024 | >1,000 | 5 | google play, app store | Education app, Supporting app |
| 118 | React Mobile Hospitality | North America | 2022 | 2024 | >1,000 | unclear | google play, app store | Emergency app, Supporting app |
| 119 | KP Women Safety app | South Asia | 2021 | 2022 | >1,000 | unclear | google play | Emergency app, Reporting and evidence building app, Supporting app |
| 120 | Balochistan Women Safety app | South Asia | 2021 | 2023 | >1,000 | 4.9 | google play | Emergency app, Reporting and evidence building app, Supporting app |
| 121 | SafeNess | Middle East & North Africa | 2023 | 2024 | >1,000 | 4 | google play, app store | Emergency app, Reporting and evidence building app |
| 122 | #NotMe | North America | 2019 | 2024 | >1,000 | 4.5 | google play, app store | Reporting and evidence building app |
| 123 | SABRE Personal Safety | North America | 2020 | 2023 | >1,000 | 3.1 | google play, app store | Emergency app, Avoidance app |
| 124 | Vault Platform | Global | 2019 | 2024 | >1,000 | unclear | google play, app store | Reporting and evidence building app |
| 125 | STOPit Notify | North America | 2021 | 2024 | >1,000 | 4.1 | google play, app store | Emergency app, Reporting and evidence building app |
| 126 | Chapperone | Global | 2020 | 2024 | >1,000 | unclear | google play, app store | Emergency app, Avoidance app |
| 127 | myPlan app | North America | 2022 | 2024 | >1,000 | 4.2 | google play, app store | Reporting and evidence building app, Supporting app |
| 128 | SafeU | North America | 2021 | 2023 | >1,000 | unclear | google play, app store | Emergency app, Reporting and evidence building app, Supporting app, Avoidance app |
| 129 | uSafeUS | North America | 2016 | 2024 | >1,000 | 4.1 | google play, app store | Emergency app, Avoidance app |
| 130 | Ride Safe | Middle East & North Africa | 2023 | 2024 | >1,000 | unclear | google play, app store | Avoidance app |
| 131 | POWA GBV | Sub-Saharan Africa | 2016 | 2017 | >1,000 | unclear | google play | Avoidance app, Reporting and evidence building app, and Supporting app. |
| 132 | Joy (জয়) | South Asia | 2020 | 2020 | >1,000 | 4.6 | google play | Emergency app, Reporting and evidence building app |
| 133 | Path - Community Safety | Europe & Central Asia | 2021 | 2023 | >1,000 | 4.8 | google play, app store | Avoidance app |
| 134 | Caminamos Juntas | Latin America & Caribbean | 2022 | 2024 | >1,000 | 3.9 | google play, app store | Reporting and evidence building app |
| 135 | Defensive Women | Europe & Central Asia | 2022 | 2023 | >500 | unclear | google play | Emergency app, Supporting app |
| 136 | Pormi | Europe & Central Asia | 2017 | 2017 | >500 | unclear | google play | Education app, Reporting and evidence building app, Supporting app |
| 137 | ASK DC | North America | 2015 | 2019 | >500 | unclear | google play | Emergency app, Supporting app |
| 138 | Companion Mobile Safety | North America | 2020 | 2024 | >500 | 5 | google play, app store | Emergency app, Avoidance app |
| 139 | RESPOND Lebanon | Middle East & North Africa | 2017 | 2023 | >500 | unclear | google play, app store | Education app, Supporting app, |
| 140 | Copwatch Panic app | Sub-Saharan Africa | 2020 | 2024 | >500 | unclear | google play, app store | Emergency app |
| 141 | Women Security - Security app | North America | 2023 | 2023 | >100 | 5 | google play | Emergency app, Supporting app |
| 142 | Watch Over Me | East Asia & Pacific | 2020 | 2024 | >100 | unclear | google play, app store | Emergency app, Avoidance app |
| 143 | SafeTrek-Protect what matters | East Asia & Pacific | 2024 | 2024 | >100 | unclear | google play, app store | Avoidance app, Reporting and evidence building app |
| 144 | GVA Ni mas Ni menos | Europe & Central Asia | 2016 | 2016 | >100 | unclear | google play | Education app, Supporting app, Avoidance app |
| 145 | bMOREsafe | North America | 2019 | 2024 | >100 | unclear | google play, app store | Avoidance app, Supporting app |
| 146 | iCut | Sub-Saharan Africa | 2020 | 2022 | >100 | unclear | google play | Education app, Supporting app |
| 147 | Safetrac InstAid | Sub-Saharan Africa | 2021 | 2024 | >100 | unclear | google play, app store | Emergency app |
| 148 | নারী সুরক্ষা Stri Suraksha | South Asia | 2017 | 2023 | >100 | unclear | google play | Education app, Reporting and evidence building app, Supporting app |
| 149 | Panic Protect | Sub-Saharan Africa | 2020 | 2024 | >100 | 5 | google play, app store | Emergency app |
| 150 | SRU Panic app | Sub-Saharan Africa | 2020 | 2024 | >100 | unclear | google play, app store | Emergency app |
| 151 | U ASK Washington, DC 2.0 | North America | 2015 | 2019 | >100 | unclear | google play | Emergency app, Supporting app |
| 152 | ASK AZ | North America | 2015 | 2019 | >100 | unclear | google play | Education app, Supporting app |
| 153 | Rescuer | Europe & Central Asia | 2019 | 2019 | >100 | unclear | google play | Emergency app |
| 154 | fightback | South Asia | 2024 | 2024 | >50 | unclear | google play | Education app |
| 155 | AnaBella app | Europe & Central Asia | 2020 | 2023 | >50 | unclear | google play, app store | Supporting app |
| 156 | SAFEsnap | North America | 2022 | 2022 | >50 | unclear | google play, app store | Emergency app, Supporting app |
| 157 | UASK Hurst | North America | 2019 | 2019 | >50 | unclear | google play | Supporting app, Reporting and evidence building app |
| 158 | 安全 Ānquán-Pak-China | South Asia | 2024 | 2024 | >10 | unclear | google play | Emergency app, Avoidance app, Supporting app |
| 159 | UASK DMV | North America | 2018 | 2019 | >10 | unclear | google play | Emergency app, Supporting app, Reporting and evidence building app |
| 160 | ASK CT | North America | 2015 | 2019 | >10 | unclear | google play | Emergency app, Supporting app |
| 161 | ASK ND | North America | 2015 | 2019 | >10 | unclear | google play | Emergency app, Supporting app |
| 162 | SAJACC Mitra | South Asia | 2023 | 2023 | >5 | unclear | google play | Emergency app, Avoidance app, Reporting and evidence building app, Supporting app |
| 163 | GWEN Alert | North America | 2019 | Unclear | >3 | unclear | google play | Emergency app |
| 164 | Personal Safety | Global | 2020 | 2024 | >1 | 4.2 | google play | Supporting app, Emergency app |
| 165 | For Her: Women Safety app | South Asia | 2023 | 2023 | >1 | unclear | google play | Emergency app, Education app |
| 166 | VictimsVoice (Web) | North America | 2017 | Unclear | Unclear | unclear | Web | Reporting and evidence building app, Supporting app |
| 167 | Arc | East Asia & Pacific | 2019 | 2019 | Unclear | unclear | app store | Reporting and evidence building app |
| 168 | Waltham Forest Safe Streets | Europe & Central Asia | 2022 | 2022 | Unclear | unclear | app store | Reporting and evidence building app, Supporting app |
| 169 | Here for You | North America | 2013 | 2020 | Unclear | 5 | app store | Supporting app, Education app |
| 170 | SirenGPS | Global | 2016 | 2019 | Unclear | 4.3 | app store | Emergency app |
| 171 | SMART24 - Keeping you safe | Middle East & North Africa | 2018 | 2024 | Unclear | 4.3 | google play, app store | Emergency app |
| 172 | Vismo GPS Tracker | Global | 2020 | 2024 | Unclear | 3 | app store | Emergency app, Reporting and evidence building app |
| 173 | invisaWear | North America | 2018 | 2023 | Unclear | 4.3 | app store | Emergency app, Avoidance app, Supporting app |
| 174 | Family Locator by Fameelee | Europe & Central Asia | 2017 | 2022 | Unclear | 4.1 | app store | Avoidance app, Reporting and evidence building app |
| 175 | Every Woman Treaty | Global | 2019 | 2021 | Unclear | 5 | app store | Education app, Supporting app |
| 176 | SEND HELP - SOS Panic Button | Global | 2012 | 2017 | Unclear | 3.1 | app store | Emergency app |
| 177 | Juntas | Global | 2016 | 2018 | Unclear | unclear | app store | Education app, Supporting app |
| 178 | Domestic Abuse | Latin America & Caribbean | 2014 | Unclear | Unclear | Unclear | microsoft store | Education app |
